# Determination and Comparison of Fat and Fibre Contents in Gluten-Free and Gluten-Containing Flours and Breads: Nutritional Implications

**DOI:** 10.3390/foods14050894

**Published:** 2025-03-05

**Authors:** María Purificación González, Paloma López-Laiz, María Achón, Rocío de la Iglesia, Violeta Fajardo, Ángela García-González, Natalia Úbeda, Elena Alonso-Aperte

**Affiliations:** Food and Nutrition in Health Promotion Research Group (CEU-NutriFOOD), Departamento de Ciencias Farmacéuticas y de la Salud, Facultad de Farmacia, Universidad San Pablo-CEU, CEU Universities, Urbanización Montepríncipe, 28660 Boadilla del Monte, Spain; p.lopez83@usp.ceu.es (P.L.-L.); achontu@ceu.es (M.A.); rocio.delaiglesia@ceu.es (R.d.l.I.); violeta.fajardomartin@ceu.es (V.F.); angargon@ceu.es (Á.G.-G.); nubeda@ceu.es (N.Ú.); eaperte@ceu.es (E.A.-A.)

**Keywords:** gluten-free, gluten-containing, flour, bread, fat, fibre

## Abstract

The absence of gluten is a technological challenge that requires the addition of components to replace the unique viscoelastic properties of gluten, thus altering the nutritional composition of gluten-free (GF) breads. Moreover, GF flours may have different compositions as compared to gluten-containing (GC) counterparts because of a different origin. This may impact the nutritional quality of GF diets. The aim of the study is to provide updated analytical data on moisture, fat, and fibre contents in GF flour and bread samples, and compare them with their GC counterparts, as well as to analyse ingredients and how they impact nutritional quality. A total of 30 different flours and 24 types of bread were analysed using AOAC methods. GF cereal flours contain more fat than GC flours (3.5 ± 2.1% vs. 2.5 ± 2.1%, *p* < 0.001), as well as GF flours from pseudocereals, except for wholemeal buckwheat (2.6 ± 0.1%). Fibre content is lower in GF flours (3.6 ± 3.1% vs. 7.1 ± 3.9%, *p* = 0.03), except for GF pseudocereal and legume flours. GF breads contain almost twice as much fat 6.6 ± 2.3% vs. 1.4 ± 0.2%, *p* < 0.001, and 4.2 ± 1.2%, *p* < 0.001) and fibre (7.3 ± 2.4% vs. 2.8 ± 0.5%, *p* < 0.001, and 4.9 ± 2.1%, *p* = 0.002) as GC breads. This is due to the raw materials themselves and to the addition of ingredients, such as regular and high oleic sunflower oil, and psyllium. Fibre ingredients and additives are more frequently used in ready-to-eat GF flours and breads, and more GF breads also contain fat-based ingredients, as compared to GC. Amaranth and chickpea flours are good alternatives to produce breads with better nutritional quality. Analysis of GF products for critical nutrients is peremptory because of continuing technological and nutritional innovation.

## 1. Introduction

Coeliac disease (CD) is characterised by inflammatory damage to the small intestine when genetically predisposed individuals consume gluten [1,2]. The pooled global seroprevalence of CD is 1.4%, while in Spain it is between 0.2% and 0.8%, and there is evidence of an increase over time [3]. However, several studies have pointed out that many patients remain undiagnosed [4,5,6].

Gluten refers to the water-insoluble proteins present in wheat, rye, and barley, and is frequently used as an ingredient in processed foods, to improve texture, moisture retention and flavour [7]. Proteins present in gluten contribute to the rheological properties of dough, and especially, the viscoelastic property: sequestration of carbon dioxide that is released during bread fermentation [7,8].

Even though the sensitivity to gluten varies greatly between patients, and most patients can tolerate small amounts of gluten in their diet [2], a gluten-free (GF) diet is the only treatment for CD and consists of strictly and permanently eliminating gluten from the diet. Since it is a lifetime requirement, the GF diet must be pleasant, sufficient, and most importantly, balanced; i.e., it must meet the individual’s nutritional needs and contain optimum amounts of macro- and micronutrients [9,10].

Nevertheless, it is difficult to follow a strict GF diet in the Western world, as wheat is one of the most consumed and used cereals [9,10]. Moreover, compliance may be challenging as patients may feel that certain food groups are eliminated from their daily diet, i.e., pasta, bread, or cakes [4,9]. Additionally, gluten may be used as an ingredient, and can, therefore, be present in unsuspected products, like processed meat or reconstituted seafood, among others [7].

Moreover, in recent years, there has been a trend of people without CD switching to the GF diet, due to a perception of increased healthiness. This shift is disturbing, since it may pose several risks, such as an increased risk of eating disorders because of exclusion, or the possibility that the diet may not be nutritionally adequate due to the intake of processed GF products [11,12].

According to the Commission Implementing Regulation (EU) No 828/2014 of 30 July 2014 [13], a product can be designated GF if it contains less than 20 mg/kg of gluten. In addition, there are so-called very low gluten foods, which are products that consist of GC cereals, but whose gluten content has been lowered to 100 mg/kg [13].

GF foodstuffs replace staple products, such as bread or pasta, and thus become a major component of diets [12]. Consumption of these products contributes at least a quarter of total calorie intake in Spanish children and adolescents, indicating their significant impact on diet and nutritional balance [14]. Therefore, patient organisations such as the Federation of Associations of Coeliacs of Spain (FACE) do not recommend basing the diet of people with CD on GF products [15].

The absence of gluten in a dough makes it less elastic and more challenging to work with [16]. In general, GF products stale quickly, which implies a loss of aroma, reduced elasticity and softening of the crust [17]. In addition, they have a texture that crumbles more easily [16,17]. The GF industry has therefore focused on increasing the shelf life of products and improving their rheology, which have indirectly affected their composition, and hence, their nutritional characteristics [17]. For example, various studies have reported a higher fat and lower protein content in GF bread [12,18,19]. Given that every Spaniard consumed 27.35 kg of bread in Spain in the year 2023 [20], these differences can have a major impact in the long run.

Several studies have addressed the disparities in composition between GF and GC products available in different countries. In the bread subcategory, GF products have been found to contain from 1.1 to 1.3 more total fat compared to their GC counterparts [21,22]. In the UK, Fry et al. [23] also describe a higher proportion of GF breads containing more fat and less fibre and protein as compared to GC, but no significant differences in the case of flours. On the other hand, other studies in Saudi Arabia [24] and Spain [25] did not find significant differences in total fat content in GF breads but a higher content of saturated fat.

GF products are a target for innovation [12] and, in fact, reformulation of some of the products is taking place [26]. For example, earlier studies indicated that CD patients consumed too little fibre [27,28,29], partly because GF products contained decreased levels of this nutrient [30]. However, dietary fibres (e.g., psyllium and bamboo fibre) and hydrocolloids have been increasingly introduced in GF products for their technological properties: they develop a stabler structure and reduce staling rate [17]. In fact, recent studies have found that GF products generally contain good quantities of fibre [31,32], which has impacted the diet patterns of CD patients, as they now consume the same amount as people without the disease [33,34].

Nonetheless, most of the articles on the composition of GF products have been carried out using the information on the nutritional label [23,24,25], especially those comparing GF and GC products. Studies based on labels usually include an elevated number of samples (+100), but from different food groups, thus narrowing the analysis of specific types like flours or breads. Studies based on analytical data are scarcer [21,35] and the number of samples is usually limited (<20), due to research impediments. Additionally, the food industry is continuously innovating and launching new products, thus, a need for further studies on GF food composition.

## 2. Materials and Methods

### 2.1. Labelling Information

All products were photographed to create a database with the information contained on their packaging, including the following:

- Nutritional information: calories, total fat, saturated fat, total carbohydrates, sugars, protein, and salt per 100 g. In addition, if the labels presented information on the quantities of other types of fat, starches, fibre, or vitamins and minerals, they were also compiled;
- Ingredient list;
- Claims: nutritional, health, and disease reduction, or other;
- Information on the source from which the product was obtained.

### 2.2. Materials and Sample Preparation

The experimental samples were flour and bread. Prior to their purchase, a study of current legislation [36,37] and consumption of these foods was carried out. Thus, a list was created with the types of flour and bread to be purchased, including three brands for each type, except for those where only two brands were found. Brands were selected according to their market share. The intention behind using three products from different brands is to minimise the impact that the use of ingredients may have on the nutritional composition of the product (method recommended by the FDA [38]). Samples were collected by convenience sampling assuming the limitations of this method.

Foods were purchased from hypermarkets such as Carrefour, Alcampo, Hipercor, Mercadona, Aldi and Lidl, as well as from retail shops specialising in GF products (e.g., Maná Productos Sin Gluten). For items not available from these sources, online suppliers were used. GF products were all labelled with the Crossed Grain symbol that certifies the product is safe to use by consumers with CD.

Flour and bread samples were prepared based on the Association of Official Agricultural Chemists (AOAC) International methods 925.08 [39] and 926.04 [40], respectively, adapted according to the FAO [41] food sample preparation methodology.

**Figure 1:**
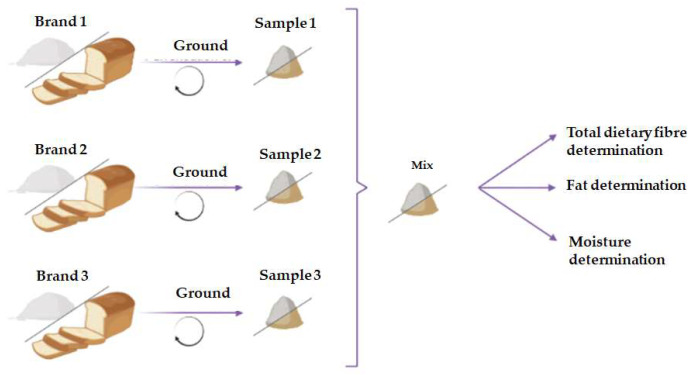
Preparation procedures of Flour and bread samples.

To obtain a single and representative sample of each type of flour or bread, a mixing process of the three brands was carried out. A diagram of this sampling protocol is shown in Figure 1, and the description is given below:

1. Flours: A total of 25 g of each brand were pooled and mixed to obtain a homogeneous sample;
2. Breads: A piece of the middle part from common and special breads of each brand was grounded by two 20 s grinds at 3100 rpm. In the case of crisp breads, random pieces were ground twice for 20 s at 5800 rpm. The grounding process was performed with the Thermomix TM31-1 (Vorwerk, Germany). Then, 25 g of each brand was pooled to obtain the final sample. When only two brands were obtained, 35 g of each brand was collected.

### 2.3. Total Dietary Fibre Determination

Total Dietary Fibre was determined using the Association of Official Agricultural Chemists (AOAC) International method 985.29 [42]. Briefly, this procedure consisted of enzymatic hydrolysis of the sample after an overnight drying process using amylase, protease, and amyloglucosidase, followed by fibre precipitation using ethanol. Precipitates are then washed with different solutions of increasing hydrophobicity, and protein and ash contents are determined. Final fibre content was given by weight (g/100 g) of residue after subtraction of protein and ash contents, as well as contribution of reagents to the residue. Enzymes were provided by the TDF-100A kit from Sigma, USA. The process was carried out on the CSF 6 Filtration System (VELP Scientifica, Usmate, Italy).

Protein content in fibre precipitates was analysed according to the Kjeldahl nitrogen analysis as specified in the AOAC method 960.52 [43]. This process consisted of transforming all organic nitrogen into inorganic nitrogen for subsequent determination by titration with HCl of the standard concentration. Ash content in fibre precipitates was determined after placing the samples for 5 h in a muffle furnace at 525 °C as specified in the AOAC method 930.22 [44]. The contribution of reagents to the precipitate was determined using blanks.

### 2.4. Fat Determination

The fat content of the samples was determined through the AOAC method 922.06 [45]. The procedure begins with fat hydrolysis by heating to 150 °C in the presence of a strong acid, specifically hydrochloric acid. This process facilitated the release of fats from the organic matrix, ensuring effective extraction of bound lipids and preventing underestimation of total fat content. Samples were then filtered in double filter funnels and washed with distilled water until the pH of the filtrate was close to neutral. Finally, total fat was extracted through the Soxhlet method using petroleum ether as an extracting solvent. A Soxtec extractor (Foss Tecator, Höganäs, Sweden) was used.

### 2.5. Moisture Determination

Moisture in the samples was determined through the AOAC method 925.09 [46]. It consists of weighing triplicates of the sample into capsules of known weight and placing them in an oven at 100 °C for 5 h. A preliminary weight measurement was taken at room temperature. The sample was then placed in the oven for an additional hour to confirm the constant weight. Prolonged heating may cause loss of volatile compounds, and sample degradation, leading to an underestimation of moisture content.

### 2.6. Statistical Analysis

Descriptive data on ingredients is expressed as frequency (number of products including a specific ingredient and percentage based on the total products within the category or the subgroup). Data on nutrient composition is expressed as average together with standard deviation, median and interquartile range. Differences between GF and GC flours and breads were tested using Student’s *T*-test, and Mann–Whitney U test in the case of non-parametric variables. The assumptions of independence, normality and homogeneity of variances of variables for Student *T*-tests and the appropriateness of the Mann–Whitney U-test were analysed and confirmed before selecting the applicable test. The statistical analysis was performed using IBM SPSS Statistics for Windows, version 27.0 (IBM Corp, Armonk, NY, USA, EE.UU.). The level of significance was set at *p* < 0.05.

## 3. Results

A total of 21 different flours, 9 ready-to-use flour mixes, and 24 different breads were analysed; 50% were GC products, and 50% were GF. For the most part of the products, three different brands were selected, thus accounting for a total of 159 different food products.

### 3.1. Flour and Ready-to-Use Flour Mixes

#### 3.1.1. Labelling Information

**Figure 2:**
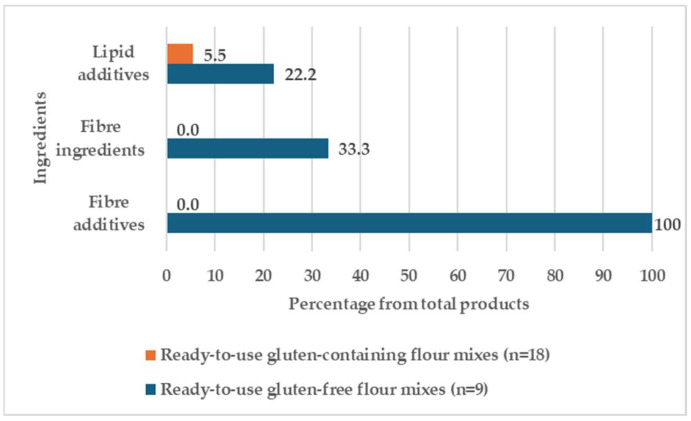
Frequency of use (%) of fat and fibre ingredients in the formulation of ready-to-use gluten-containing and gluten-free flour mixes. Expressed as a percentage based on the total number of products within the category or the subgroup.

Most flour samples contain a single ingredient, which consists of the flour of the cereal, pseudocereal, or legume in question, or a mix of multigrain flours, but ready-to-use flour mixes include a mix of flours, non-cereal ingredients, and different kinds of additives (mostly emulsifiers and thickeners). Table 1 highlights the types of fibre and fat ingredients used in the formulation of GC and GF ready-to-use flour mixes, while whole ingredient lists are presented in Supplementary Tables S1 (GC flours), and S2 (GF flours). The frequency of use (%) of fat and fibre ingredients in the formulation of ready-to-eat flour mixes GC and GF is shown in Figure 2.

As for fibre, both ingredients containing fibre and fibre additives are considered. Ingredients that provide fibre (not additives—fibre) are those that provide fibre naturally and constitute part of the food, for example, wheat bran in ciabatta bread. These ingredients not only provide fibre but also other nutrients and desirable characteristics of the food. Fibre additives are usually extracted or synthesised fibres that are added in concentrated form to improve certain properties such as texture or water retention capacity, in breads. In short, the total fibre in a food can come from ingredients that provide fibre and additives that are fibre. Nonetheless, a fibre ingredient may be considered both a food ingredient and an additive, as is the case for xanthan gum.

None of the samples of the GC ready-to-use flour mixes contain fibre ingredients or additives; in contrast, the GF ready-to-use flour mixes do. All GF ready-to-use flour mixes include some sort of added fibre, with gums being the most frequently used additive (66.7%). Vegetable fibres are used in the formulation of bread mixes, and other products contain cellulose derivatives, pectin, or alginate.

Regarding the use of lipids, both types of samples contain only lipid additives. These are more frequently present in GF ready-to-use flour mixes (22.2% of GF products vs. 5.5% of GC products) and are only found in one GC sample: the bread mix. Surprisingly, GC bread mix contains lipid additives, but GF bread mix does not.

#### 3.1.2. Analytical Determinations

**Figure 3:**
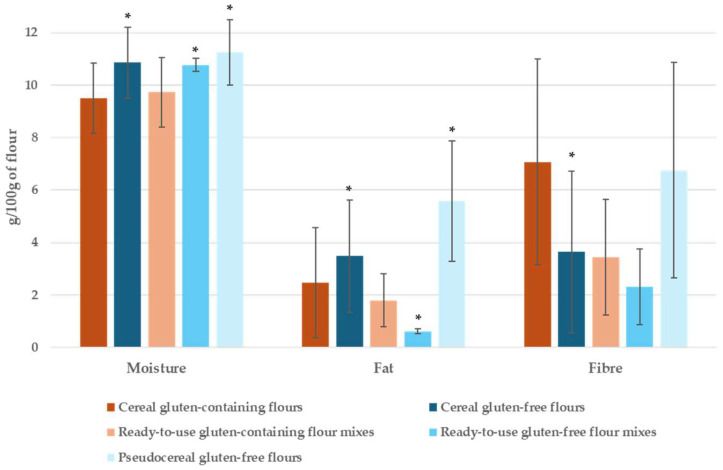
Average moisture, fat and fibre contents in gluten-containing and gluten-free flours. (*) *p* > 0.05 GC vs. GF.

The analytical data regarding moisture, fat, and fibre contents in the flour samples are shown in Table 2. The average results are represented in Figure 3. Moisture content varies greatly between the samples (from 7.5% to 16.1%), probably due to the different ingredient formulations (Supplementary Tables S1 and S2).

Regarding fat content, several findings are remarkable. Firstly, it is surprising that there is such a significant and large (1.4-fold) difference between GC and GF (2.47 g/100 g vs. 3.48 g/100 g, *p* < 0.001) cereal flours, with the GF providing a higher amount of fat (Table 2). On the other hand, the fat content of GF ready-to-use flour mixes is significantly lower than the fat content of all GC ready-to-use flour mixes (0.62 g/100 g vs. 1.80 g/100 g, *p* < 0.001), and they have a different ingredient profile (Supplementary Tables S1 and S2). Nonetheless, when preparing products from ready-to-use flour mixes, further ingredients such as oil, butter or eggs may have to be added, thus providing an extra amount of fat.

Secondly, it is noteworthy that both samples of whole grain oat flour (GC and GF) contain a very high content of fat, which is approximately double that of the samples with the second highest amount of fat.

Flours from pseudocereals such as amaranth and quinoa contain higher amounts of fat, as compared to cereals, both GF and GC (5.58 g/100 g vs. 3.48 g/100 g, *p* = 0.06; 5.58 g/100 g vs. 2.47 g/100 g, *p* < 0.001), except for buckwheat flour, which contains an amount of fat close to cereals such as sorghum or teff.

Potato starch has a completely different fat content compared to other flours (cereals (3.48 g/100 g), pseudocereals (5.58 g/100 g), or legumes (4.46 g/100 g), but similar to that of GF ready-to-use flour mixes (0.62 g/100 g) for bakery, pizza and all-purpose uses. This is not surprising, as all these products contain a wide variety of starches [47]. Finally, wholemeal versions of the flours, both GC and GF, have a higher fat content as compared to their refined options. This arises because of the grain being milled whole and the bran and the germ providing a considerable amount of fat [48]. In the case of some GF flours, the package does not indicate whether the product is wholemeal or not, i.e., teff flour, but its fibre content may indicate that it is in fact wholemeal.

The GC flour sample with the highest amount of fibre is wholemeal rye flour, closely followed by wholemeal wheat flour. In contrast, the two GF samples with the highest value are not wholemeal flours, but amaranth and chickpea, i.e., from a pseudo-cereal and a legume, respectively. This illustrates that there is no need to be limited to cereals to find sources of fibre. In fact, the lowest value for fibre in a GF sample is from a cereal, sorghum, which contains an even slightly lower fibre amount than potato starch, which is composed entirely of starch (0.36 g/100 g vs. 0.40 g/100 g, respectively).

GF cereal and pseudocereal flours tend to have a higher moisture content than GC flours (*p* < 0.002). As compared to GC cereal flours, GF legume flour provides a significantly higher amount of fat (*p* < 0.05) and more fibre (n.s.).

### 3.2. Bread Samples

#### 3.2.1. Labelling Information

In the following Table 3, Table 4, Table 5, Table 6 and Table 7, breads are classified according to the Spanish regulation [37] as common or special. In the norm, bread, without any other qualifier, is defined as the product resulting from the cooking of dough obtained by mixing flour and water, with or without the addition of salt, fermented with the help of baker’s yeast or sourdough. In addition, other ingredients listed in this standard may be incorporated into the bread dough. Common bread is fresh bread, baked on the same day and sold unpacked. It may include cereal bran as an ingredient. Special breads are all those that differ from common bread because of composition or because of the making process. Differences in composition include the use of prepared flour, i.e., GF wheat flour, or the addition of further ingredients and additives. Bread is also considered special if grated, baked in a mould, with special shapes or with partial blanching of the flours, among others. Bread may also be twice baked or dried for preservation. That is the case for rusks, breadsticks, and breadcrumbs, which are all considered special breads.

Table 3 and Table 4 show the addition of lipid ingredients in the bread samples. In this case, 93.9% of the GF breads contain some fat-based ingredients, while these are only found in 63.9% of the GC breads. In contrast, the presence of lipid additives is approximately equal in both groups (47.2% GC, 54.5% GF).

Except for the presence of fat additives, common GC breads do not contain any source of fat. On the other hand, special breads tend to contain both lipid-rich ingredients and fat-based additives, whether they are GC or GF breads. The most frequently used lipid ingredient in both types of samples is sunflower oil (47.2% of GC bread, 54.5% of GF bread), followed by extra virgin olive oil in 15.2% of GF bread, and olive oil (11.1% of GC bread). Therefore, due to the composition of these oils, the lipid profile of these products consists of a higher proportion of unsaturated fatty acids [47]. However, other fatty ingredients rich in saturated fatty acids, such as margarine, coconut, and palm oils, are only found in GF breads, albeit in very low proportions.

The results of the study of fibre sources in breads are given in Table 5 and Table 6.

Firstly, it is remarkable but understandable that both wheat bran and cereal fibres (from wheat and oats) are only found in breads containing gluten since these fibres contain protein. On the other hand, 72.7% of the GF breads include vegetable fibres, while only 2.8% of the GC breads do. This huge difference is also found when comparing the presence of the different additives: while 54.5% and 78.8% of GF breads include gums and cellulose derivatives as additives, respectively, only 11.1% of the GC samples contain gums, and none of them contain cellulose derivatives. Furthermore, all GF foods contain fibre additives, whereas these are only found in 8.3% of GC breads. Finally, the frequency of the presence of some type of fibre as an ingredient in the GC breads is 25.0%, being more than three times higher in the GF samples (75.8%).

It is noticeable that GC crisp breads do not contain any ingredients or additives as a source of fibre. In contrast, 100% of this type of GF breads contain additives such as thickeners and emulsifying agents and more than half of the samples (55.6%) also include fibre. Another group of breads worth mentioning are the common GC breads, in which only 11.1% contain fibre, in this case, wheat bran.

**Figure 4:**
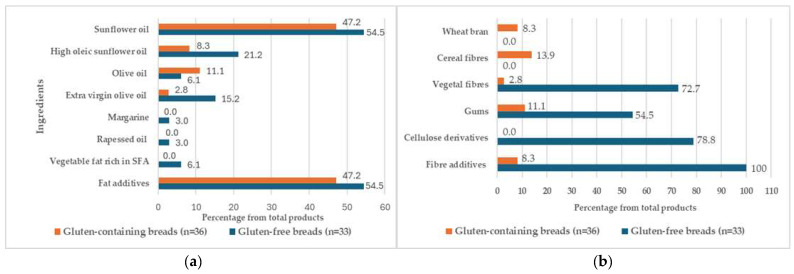
Frequency of use (%) of fat (**a**) and fibre (**b**) ingredients in the formulation of gluten-containing and gluten-free breads. SFA: saturated fatty acid.

The frequency of use (%) of fat and fibre ingredients in the formulation of GC and GF breads is shown in Figure 4. Other ingredients besides fat components and fibres found in GC and GF breads are described in Supplementary Tables S3 and S4.

#### 3.2.2. Analytical Determinations

Table 7 shows the analytical data for moisture, fat, and fibre contents in the bread samples. Regarding moisture content, two findings stand out. Firstly, and surprisingly, all GF breads contain a higher proportion of moisture compared to their GC counterparts. Secondly, a clear difference is observed between the moisture values of common breads and special breads, including dry or crisp breads, with the latter consistently displaying lower moisture values. Furthermore, even though both GC and GF crisp breads have lower levels of moisture compared to the rest, significant differences are also found between them. In this case, GF samples also have higher moisture content than their GC equivalents.

When studying fat content values, it is evident that all GF groups show statistically significant differences compared to their GC counterparts, with the GF sample always having a higher content. For example, the GF hot dog buns contain more than twice as much fat as their GC counterparts (3.66 g/100 g GC, 6.34 g/100 g GF, *p* < 0.001). The only sample that does not follow this trend is the GF loaf, as it has less fat than the regular version.

A surprising finding is found when comparing seeded or multigrain breads with white sliced bread: while the GC seeded breads contain almost twice as much fat as white bread (GC seeded loaf: 5.25 g/100 g, GC white sliced bread: 2.68 g/100 g, *p* < 0.001), the GF samples are not so far from each other, in the case of multigrain loaf bread (GF multigrain loaf bread: 7.98 g/100 g, GF white sliced bread: 8.27 g/100 g *p* = 0.014) or in the case of seeded sliced bread (GF seeded sliced bread: 7.52 g/100 g, GF white sliced bread 8.27 g/100 g *p* = 0.025). Since seeded or multigrain breads contain whole grains, which are rich in fat, it is not surprising that their fat levels are higher. As white sliced bread does not contain such seeds, its GF version must contain a large amount of extra fat to explain similar levels. According to Table 4, all these samples contain fat as an ingredient, but the amount added is not reported.

The results for fibre are also remarkable: when making comparisons between equivalent groups, the GF product has a higher fibre content in all but two cases, i.e., the loaf and the breadcrumbs.

The GC bread with the highest amount of fibre is the one made with refined rye flour. Since our results in Table 2 show that wholemeal rye flour has the highest fibre content, the refined version contains probably more fibre than the refined wheat flour and, therefore, rye bread has more fibre than white bread.

**Figure 5:**
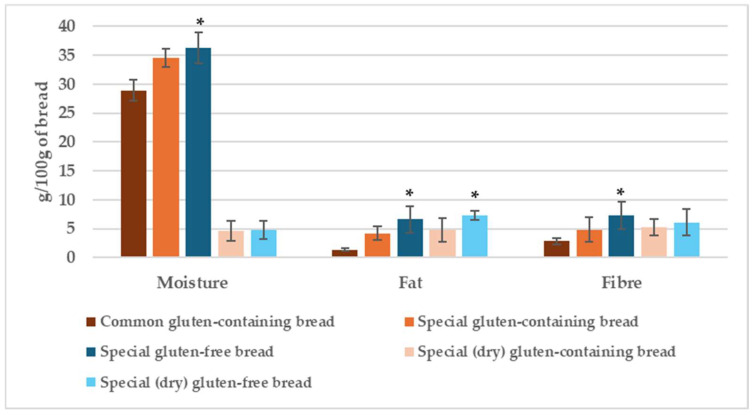
Average moisture, fat and fibre contents in gluten-containing and gluten-free bread. (*) *p* > 0.05 GC vs. GF.

Average values for analytical data on moisture, fat, and fibre contents of GF and CG breads are presented in Figure 5.

In summary, cereal and pseudocereal GF flours contain as much as 1.4-fold more fat than GC cereal flours, except for buckwheat. Flours from pseudocereals and legumes, i.e., amaranth and chickpea, provide higher amounts of fibre as compared to refined GC cereals, but other GF flours are generally low in fibre. In the case of breads, fat content is also significantly higher in GF samples, especially when compared to common GC bread. Finally, the study of ingredients proved that the addition of fibre ingredients and additives is common in GF ready-to-use flour mixes and breads, thus rendering an unexpectedly high content of fibre in these products. In the case of GF breads, 93.9% of the samples also included fat additives, most frequently sunflower and olive oils.

## 4. Discussion

Analysing the nutritional composition of GF flours and breads is an important issue nowadays because they are staple foods for children and adults with celiac disease. Adults on a GF diet depend to a lesser extent on GF commercial products, but children and adolescents consume GF products two to three times a day, accounting for as much as 165 g/day [49]. In Spanish children and adolescents, GF products contributed with a high percentage (>25%) to total energy, carbohydrates, fibre, and salt daily intakes and, to a lesser extent (<20%), to fat (including saturated fat), sugars and protein [49]. They also contribute significantly to a balanced breakfast for children with CD [50]. Moreover, there has been a great improvement, thanks to technological innovations, in the availability, diversity, and acceptability of these products and, consequently, consumption is rising even in the population without celiac disease. Once technological issues have been overcome, it is time for nutritional improvement.

Taken together, our results for GF flours and ready-to-use flour mixes show that GF flours contain more fat, less fibre, and significantly higher levels of moisture than GC flours.

GF flours may contain as much as 1.4-fold more fat than GC cereal flours, being oat flour the one with the highest fat content, followed by pseudocereals such as quinoa and amaranth, legume flours, and buckwheat. In the case of pseudocereals, while the fat contents of amaranth and quinoa do not differ significantly from each other, that of buckwheat does, being much lower and closer to that of cereals such as sorghum or teff. In fact, pseudocereals such as buckwheat have been proposed as useful in GF bread formulation [51]. The 50% replacement of potato starch by milled amaranth, quinoa, and buckwheat pseudocereals in GF baking provides breads with higher levels of protein, fat, fibre, and minerals [52]. Moreover, fat content in pseudocereals is described as highly unsaturated, with the highest unsaturated/saturated ratio observed from quinoa [35,52].

GF flours are also low in fibre. When comparing the two types of flour most used in bread formulation, i.e., wheat flour for GC breads and rice flour for GF samples, it can be concluded that, although the difference is not statistically significant, rice flour tends to have slightly less fibre. Furthermore, it is also surprising to see that there is no difference between GF rice flour and its wholemeal equivalent. Instead, a clear contrast can be observed between GC wheat flour and its wholemeal version. Therefore, a product using wheat flour as the main ingredient should have more fibre than a GF product made with rice flour, especially if the base of the GC product is whole wheat. Using flours from legumes or from pseudocereals such as amaranth in GF product formulation could improve the final fibre content. The highest contents for fibre were found for amaranth and chickpea flours. Legume flours are important sources of nutrients such as proteins, complex carbohydrates, fibres, micronutrients, and antioxidant compounds, and could also improve the nutritional quality of GF products [53,54].

Moisture content varies greatly between the samples (from 7.5 to 16%), probably due to the different ingredient formulations (Supplementary Tables S1 and S2). In fact, all the ready-to-use GF flour mixes contain different starches, which are hygroscopic ingredients that retain moisture [55]. The sample with the highest moisture content is potato starch (16.13 g ± 0.08). On the other hand, chickpea flour presents the lowest level (7.84 g ± 0.11), due to naturally containing less starch [56].

From a nutritional point of view, the best flours for bread formulation would be amaranth and chickpea flours. Both flours have high levels of fibre, a necessary component for a good bread structure. In addition, both also have high levels of good-quality fat, which would avoid the need to add fatty ingredients. On the other hand, and although it has not been the objective of this study, these flours also have high levels of high-quality protein [57,58].

The pattern present in GF flours (high fat and low fibre) is not repeated in breads. Here, both fibre and fat contents are higher in GF products. This result is not unexpected regarding fat, as GF flours contain more of this nutrient and fat sources are more frequently added to GF bread formulation. Results for fibre denote a significant addition of the nutrient in GF breadmaking.

GF breads contain almost twice as much fat and fibre as GC breads, mostly due to the frequent addition of fat- and fibre-based ingredients and additives. In fact, the presence of fatty ingredients and additives is important for the palatability and stability of GF products [48]. This finding is in agreement with that of Miranda et al., who states that the amount of fat in GF products is almost twice as high as in GC breads [30]. The only sample that does not follow this trend is the GF loaf, as it has less fat than the regular version. This sample varies from the others because it is presented frozen, so its composition may vary to withstand the low temperatures and be palatable once thawed. On the other hand, the GC loaf is a regular fresh bread product, which means that it must be consumed within 24 h after baking, according to Spanish Regulation [37]. Because of the different final presentation and shelf life of the products, many need to have a differing composition, resulting in nutritional variations.

Fat ingredients and additives that come into the composition of GF breads are more frequently unsaturated (sunflower oil, olive oil), but hamburger buns, breadsticks and breadcrumbs also include saturated fat from palm or coconut. Thus, fat quality in GF breads seems to be improving, probably since the European regulation [59] that introduced new requirements for food labelling, including labelling the specific vegetable origin of oils, instead of the generic term “Vegetable Oils”, and the recent trends in consumer perception against palm oil, amplified by social networks.

The introduction of fibre ingredients and additives can serve both as a technological aid [60] and to increase the amount of this type of nutrient in the breads [31], as the most used raw materials show very low fibre levels [61]. In fact, as mentioned in the introduction of the present work, there has been a shift whereby GF breads have increased their fibre content [62]. This trend is corroborated by our analytical results, as statistically significant differences are observed, with GF breads having a higher fibre content. Technological improvement goes hand in hand with nutritional improvement in the case of fibre. In fact, all the GF bread samples but one (loaf 2.61 g fibre/100 g product) can be considered as sources of fibre as they contain more than 3 g of fibre per 100 g of product. This is in line with the findings of several authors [31,32]. On the other hand, common GC breads, which are the most consumed by the Spanish population [20], contain low amounts of fibre, just over the threshold.

Added fibre in GF bread tends to be more insoluble than soluble, because of the frequent addition of cellulose additives (26 GF breads, 78.8% of samples). Nonetheless, in recent times, psyllium husk, a soluble fibre, has proven very useful in bread making, especially in GF baking. In the present study, the ingredient “Vegetal fibres” included psyllium in 18 of the GF breads (54.5%). These fibre ingredients together with xanthan gum, guar gum, and others are responsible for improving the dough structure, the tenderness of GF breads; and their shelf life, due to their hydrophilic nature [63]. These hydrocolloid compounds absorb water and form a gel-like structure, which improves dough elasticity and flexibility, which are crucial for achieving a good texture [64]. Thus, GF bread may include a healthy proportion of soluble and insoluble fibre.

GF breads have improved in fibre content, but on the other hand, because people with CD can be unaware that GF breads contain comparatively high amounts of fat, they may be consuming higher than expected amounts of fat by keeping the same intake patterns as people without CD. Therefore, GF breads would benefit from a reduction in fat to improve their nutritional quality. Recent findings by Flores and Coronel [65] suggest that the GF diet may carry a risk of lipid profile abnormalities in paediatric patients with CD that can lead to long-term metabolic issues. Angi et al. [66] reported that processed GF foods might promote unfavourable alterations of metabolic parameters, especially those associated with increased risk of cardiovascular diseases. We also reported in a female population with CD a higher percentage of body fat compared to control women [67]. Therefore, further well-designed longitudinal studies involving large numbers of people with long-term follow-up are needed to clarify whether prolonged exposure to the GF diet could result in an increased cardiometabolic risk [68]. Moreover, saturated fat ingredients and additives should also be avoided, although they would necessarily be replaced by unsaturated vegetable oils or other aids for technological reasons. The presence of saturated fat stabilises the GF product, as it is solid at room temperature [69]. Another interesting ingredient to be considered is added sugars. All GF bread samples declared added sugars in the ingredient list, while sugar is not added in common GC breads. Only GC white sliced, wholemeal sliced, and rye sliced breads, as well as hamburger buns, hot dog buns, and rusks containing added sugars.

Several studies have analysed GF product composition, mostly using declared data on food labelling [18,30,32,62] and to a lesser extent by chemical analysis [21,35]. Our results are in accordance with those of previous studies, confirming that there are some differences in the fat and fibre contents of GF flours and breads as compared to regular foods [21,30,62]. Our results for moisture, fat and fibre are in line with those published by Culetu et al. [35] for GF flours from different sources, Tres et al. [21] for fat in breads, and Alvarez-Jubete et al. [52] in the case of pseudocereals. In the case of fibre, results on GF breads are not so consistent, with some studies declaring low fibre content in GF bread [12,30] and other more recent ones declaring high contents of fibre [26,31,32]. Cornicelli et al. [62] demonstrated that GF bread has a higher fibre content with respect to GC counterparts but, conversely, GF bread substitutes (crackers and breadsticks), biscuits, and pasta, and have lower fibre contents.

## 5. Conclusions

From the results of our study, we can conclude the following:

- GF flours and ready-to-use flour mixes contain more fat, less fibre, and significantly higher levels of moisture than GC flours;
- GF breads contain almost twice as much fat and fibre as GC breads;
- Surprisingly, GF breads have significantly increased their fibre content due to the addition of fibre ingredients, indicating an improvement in their nutritional quality;
- From a nutritional point of view, GF bread composition could still benefit from the reduction in saturated fat. Using flours from pseudocereals or legumes in GF breadmaking as an alternative to GF refined cereal flours and starches should be considered.

## Figures and Tables

**Table 1:** Types of fibre and lipid ingredients used in the formulation of gluten-containing and gluten-free ready-to-use flour mixes.

	Food Subgroups (n)	Vegetal Fibres *	Additives: Gums **	Additives: Cellulose Derivatives ***	Additives: Pectin	Additives: Alginate	Fibre Additives (Total)	Fibre Ingredients (Total)	Lipid Additives ****
		n (%)	n (%)	n (%)	n (%)	n (%)	n (%)	n (%)	n (%)
**Gluten-containing flours**	Baking mix (3)	0	0	0	0	0	0	0	0
	Cake mix (3)	0	0	0	0	0	0	0	0
	Pancake and crepe mix (3)	0	0	0	0	0	0	0	0
	Frying and battering mix (3)	0	0	0	0	0	0	0	0
	Pizza mix (3)	0	0	0	0	0	0	0	0
	Bread mix (3)	0	0	0	0	0	0	0	1 (33.3)
**Gluten-free flours**	Baking mix (3)	0	3 (100.0)	0	0	0	3 (100.0)	0	1 (33.3)
	Bread mix (3)	3 (100.0)	0	3 (100.0)	0	0	3 (100.0)	3 (100.0)	0
	Multipurpose mix (3)	0	3 (100.0)	1 (33.3)	1 (33.3)	1 (33.3)	3 (100.0)	0	1 (33.3)

Results are expressed as frequency (n: number of products including a specific ingredient) and percentage based on the total products within the category or the subgroup. * Vegetal fibres comprise psyllium and bamboo, among others. ** Gums include guar gum (E412), xanthan gum (E415) and tare gum (E417). *** Cellulose derivatives include hydroxypropyl methylcellulose (E464). **** Lipid additives may include emulsifying agents E471 (mono- and diglycerides of fatty acids), E472e (mono- and diacetyl tartaric esters of mono- and diglycerides of fatty acids), E481 and E482 (sodium and calcium stearoyl-2-lactylates), and E491 (sorbitan monostearate), among others.

**Table 2:** Moisture, fat, and fibre content in gluten-containing and gluten-free flours.

		Food Subgroups (n)	Moisture (g/100 g)	Fat (g/100 g)	Fibre (g/100 g)
**Gluten-containing flours**	**Cereal flours**	Wheat (3)	10.30 ± 0.06	1.28 ± 0.01	3.74 ± 0.48
		Whole wheat (3)	9.96 ± 0.14	2.35 ± 0.20	11.06 ± 0.58
		Strong wheat (3)	8.04 ± 0.13	1.39 ± 0.05	5.20 ± 1.67
		Wholemeal spelt (3)	7.51 ± 0.12	2.52 ± 0.03	7.52 ± 0.84
		Wholemeal oat (3)	7.83 ± 0.13	8.22 ± 0.09	7.82 ± 1.24
		Wholemeal rice (3)	10.82 ± 0.06	1.47 ± 0.21	3.87 ± 0.32
		Maize (3)	11.02 ± 0.09	1.82 ± 0.03	2.22 ± 0.86
		Wholemeal rye (3)	9.30 ± 0.05	1.47 ± 0.05	14.86 ± 0.10
		Multi-grain (3)	10.72 ± 0.07	1.56 ± 0.29	7.36 ± 2.18
		**Mean ± SD** **Median (IQR)**	**9.50 ± 1.33** **9.98 (7.98–10.76)**	**2.47 ± 2.11** **1.62 (1.42–2.47)**	**7.07 ± 3.92** **6.73 (3.84–9.78)**
	**Ready-to-use flour mixes**	Baking mix (3)	11.92 ± 0.03	0.97 ± 0.11	3.40 ± 0.59
		Cake mix (3)	10.16 ± 0.17	1.34 ± 0.06	2.86 ± 1.95
		Pancake and crepe mix (3)	7.75 ± 0.01	3.94 ± 0.03	7.19 ± 5.11
		Frying and battering mix (3)	9.75 ± 0.01	1.65 ± 0.05	2.19 ± 0.90
		Pizza mix (3)	10.02 ± 0.09	1.31 ± 0.10	2.27 ± 0.61
		Bread mix (3)	8.79 ± 0.16	1.61 ± 0.02	4.05 ± 0.97
		**Mean ± SD** **Median (IQR)**	**9.73 ± 1.32** **9.84 (8.80–10.22)**	**1.80 ± 1.01** **1.51 (1.26–1.66)**	**3.45 ± 2.20** **3.12 (2.39–3.87)**
**Gluten-free** **flours**	**Cereal flours**	Maize (3)	8.57 ± 0.07	3.14 ± 0.11	2.94 ± 1.01
		Rice (3)	11.34 ± 0.04	1.50 ± 0.04	2.50 ± 0.63
		Wholemeal rice (3)	12.69 ± 0.09	2.63 ± 0.11	2.32 ± 1.58
		Wholemeal oat (3)	9.69 ± 0.06	8.30 ± 0.10	8.92 ± 0.42
		Teff (3)	10.23 ± 0.04	2.63 ± 0.02	6.17 ± 1.35
		Millet (3)	11.81 ± 0.12	3.75 ± 0.15	1.01 ± 0.29
		Sorghum (3)	11.63 ± 0.08	2.37 ± 0.02	0.36 ± 0.24
		**Mean ± SD** **Median (IQR)**	10.86 ± 1.35 * **11.33 (9.72–11.75)**	3.48 ± 2.13 * **2.65 (2.38–3.71)**	3.64 ± 3.08 * **2.94 (0.89–5.84)**
	**Pseudocereal flours**	Quinoa (3)	11.19 ± 0.04	6.34 ± 0.07	6.42 ± 0.07
		Wholemeal buckwheat (3)	12.71 ± 0.04	2.63 ± 0.09	2.20 ± 0.58
		Amaranth (3)	9.84 ± 0.02	7.75 ± 0.11	11.62 ± 0.22
		**Mean ± SD** **Median (IQR)**	11.25 ± 1.25 * **11.19 (9.85–12.70)**	5.58 ± 2.30 * **6.34 (2.68–7.69)**	**6.75 ± 4.10** **6.39 (2.52–11.49)**
	**Legume flours**	Chickpea (3)	7.84 ± 0.11	4.46 ± 1.10 *	10.87 ± 0.94
	**Starch**	Potato (3)	16.13 ± 0.08 *	0.01 ± 0.00 *	0.40 ± 0.14 *
	**Ready-to-use flour mixes**	Baking mix (3)	10.96 ± 0.08	0.69 ± 0.04	0.83 ± 0.72
		Bread mix (3)	10.45 ± 0.01	0.52 ± 0.05	2.57 ± 1.44
		Multipurpose mix (3)	10.89 ± 0.04	0.64 ± 0.07	3.54 ± 0.31
		**Mean ± SD** **Median (IQR)**	**10.76 ± 0.25#** **10.88 (10.45–10.95)**	**0.62 ± 0.09 #** **0.65 (0.55–0.70)**	**2.31 ± 1.44** **2.12 (1.17–3.68)**

Results are expressed as mean (g/100 g product) ± standard deviation (SD) and median (Percentile 25–-Percentile 75). * Cereals flours GC vs. cereals flours GF (moisture *p* = 0.002; fat *p* < 0.001; fibre *p* = 0.003). Cereals flours GC vs. pseudocereals flours GF (moisture *p* = 0.004; fat *p* < 0.001; fibre *p* = 0.701). Cereal flours GC vs. legume flours GF (moisture *p* = 0.067; fat *p* = 0.029; fibre *p* = 0.067). Cereals flours GC vs. starch GF (moisture *p* = 0.005; fat *p* = 0.005; fibre *p* = 0.005). # Ready-to-use GC flour mixes vs. ready-to-use GF flour mixes (moisture *p* = 0.005; fat *p* < 0.001; fibre *p* = 0.269).

**Table 3:** Lipid ingredients used in the formulation of gluten-containing breads.

	Food Subgroups (n)	Sunflower Oil n (%)	High Oleic Sunflower Oil n (%)	Olive Oil n (%)	Extra Virgin Olive Oil n (%)	Margarine * n (%)	Vegetable Fat Rich in SFA ** n (%)	Fat Additives *** n (%)
**Gluten-containing breads**	Loaf (3)	0	0	0	0	0	0	2 (66.7)
	Baguette (3)	0	0	0	0	0	0	2 (66.7)
	Ciabatta (3)	0	0	0	0	0	0	0
	**Total common bread (9)**	**0**	**0**	**0**	**0**	**0**	**0**	**4 (44.4)**
	White sliced (3)	2 (66.7)	0	1 (33.3)	0	0	0	2 (66.7)
	Wholemeal sliced (3)	1 (33.3)	0	2 (66.7)	0	0	0	2 (66.7)
	Rye sliced (3)	3 (100.0)	0	0	0	0	0	1 (33.3)
	Seeded loaf (3)	3 (100.0)	0	0	0	0	0	0
	Hamburger bun (3)	3 (100.0)	0	0	0	0	0	3 (100.0)
	Hot dog bun (3)	3 (100.0)	0	0	0	0	0	3 (100.0)
	**Total special bread (18)**	**15 (83.3)**	**0**	**3 (16.7)**	**0**	**0**	**0**	**11 (61.1)**
	Rusks (3)	1 (33.3)	2 (66.7)	1 (33.3)	0	0	0	1 (33.3)
	Breadsticks (3)	0	1 (33.3)	0	1 (33.3)	0	0	1 (33.3)
	Breadcrumbs (3)	0	0	0	0	0	0	0
	**Total special (dry) bread (9)**	**1 (11.1)**	**3 (3.33)**	**1 (11.1)**	**1 (11.1)**	**0**	**0**	**2 (22.2)**

Results are expressed as frequency (n: number of products including a specific ingredient) and percentage based on the total products within the category or the subgroup. * Margarine includes palm fat and sunflower oil. ** Vegetable fat rich in saturated fatty acids (SFA) includes coconut and palm oil. *** Additives include emulsifying agents E322 (sunflower lecithin), E471 (mono- and diglycerides of fatty acids), E472e (mono- and diacetyl tartaric esters of mono- and diglycerides of fatty acids), E481 and E482 (sodium and calcium stearoyl-2-lactylates), and E491 (sorbitan monostearate), among others.

**Table 4:** Lipid ingredients used in the formulation of gluten-free breads.

	Food Subgroups (n)	Sunflower Oil n (%)	High Oleic Sunflower Oil n (%)	Olive Oil n (%)	Extra Virgin Olive Oil n (%)	Margarine * n (%)	Rapeseed Oil n (%)	Vegetable Fat Rich in SFA ** n (%)	Fat Additives *** n (%)
**Gluten-free breads**	Loaf (2)	0	0	1 (50.0)	0	0	0	0	0
	Baguette (3)	0	3 (100.0)	0	0	0	0	0	2 (66.7)
	Ciabatta (2)	2 (100.0)	0	0	1 (50.0)	0	1 (50.0)	0	1 (50.0)
	White sliced (3)	2 (66.7)	1 (33.3)	0	0	0	0	0	3 (100.0)
	Seeded sliced (3)	3 (100.0)	0	0	0	0	0	0	1 (33.3)
	Multigrain loaf (3)	3 (100.0)	0	0	0	0	0	0	2 (66.7)
	Muffin (3)	2 (66.7)	0	1 (33.3)	1 (33.3)	0	0	0	1 (33.3)
	Hamburger bun (3)	2 (66.7)	0	0	0	1 (33.3)	0	0	3 (100.0)
	Hot dog bun (2)	1 (50.0)	1 (50.0)	0	1 (33.3)	0	0	0	1 (50.0)
	**Total special bread (24)**	**15 (62.5)**	**5 (20.8)**	**2 (8.3)**	**3 (12.5)**	**1 (4.2)**	**1 (4.2)**	**0**	**14 (58.3)**
	Rusks (3)	2 (66.7)	1 (33.3)	0	0	0	0	0	2 (66.7)
	Breadsticks (3)	1 (33.3)	1 (33.3)	0	1 (33.3)	0	0	1 (33.3)	1 (33.3)
	Breadcrumbs (3)	0	0	0	1 (33.3)	0	0	1 (33.3)	1 (33.3)
	**Total special (dry) bread (9)**	**3 (33.3)**	**2 (22.2)**	**0**	**2 (22.2)**	**0**	**0**	**2 (22.2)**	**4 (44.4)**

Results are expressed as frequency (n: number of products including a specific ingredient) and percentage based on the total products within the category or the subgroup. * Margarine includes palm fat and sunflower oil. ** Vegetable fat rich in saturated fatty acids (SFA) includes coconut and palm oil. *** Additives include emulsifying agents E322 (sunflower lecithin), E471 (mono- and diglycerides of fatty acids), E472e (mono- and diacetyl tartaric esters of mono- and diglycerides of fatty acids), E481 and E482 (sodium and calcium stearoyl-2-lactylates), and E491 (sorbitan monostearate), among others.

**Table 5:** Types of fibres used in the formulation of gluten-containing breads.

	Food Subgroups (n)	Wheat Bran n (%)	Cereal Fibres * n (%)	Vegetal Fibres ** n (%)	Gums *** n (%)	Cellulose Derivatives **** n (%)	Fibre Additives (Total) n (%)	Fibre Ingredients (Total) n (%)
**Gluten-containing breads**	Loaf (3)	0	0	0	0	0	0	0
	Baguette (3)	0	0	0	0	0	0	0
	Ciabatta (3)	1 (33.3)	0	0	0	0	0	1 (33.3)
	**Total common bread (9)**	**1 (11.1)**	**0**	**0**	**0**	**0**	**0**	**1 (11.1)**
	White sliced (3)	0	1 (33.3)	0	1 (33.3)	0	1 (33.3)	1 (33.3)
	Wholemeal sliced (3)	0	1 (33.3)	1 (33.3)	0	0	0	2 (66.7)
	Rye sliced (3)	1 (33.3)	0	0	0	0	0	1 (33.3)
	Seeded loaf (3)	1 (33.3)	1 (33.3)	0	1 (33.3)	0	0	2 (66.7)
	Hamburger bun (3)	0	0	0	1 (33.3)	0	1 (33.3)	0
	Hot dog bun (3)	0	2 (66.7)	0	1 (33.3)	0	1 (33.3)	2 (66.7)
	**Total special bread (18)**	**2 (11.1)**	**5 (27.8)**	**1 (5.6)**	**4 (22.2)**	**0**	**3 (16.7)**	**8 (44.4)**
	Rusks (3)	0	0	0	0	0	0	0
	Breadsticks (3)	0	0	0	0	0	0	0
	Breadcrumbs (3)	0	0	0	0	0	0	0
	**Total special (dry) bread (9)**	**0**	**0**	**0**	**0**	**0**	**0**	**0**

Results are expressed as frequency (n: number of products including a specific ingredient) and (percentage based on the total products within the category or the subgroup). * Cereal fibres include wheat and oat fibre. ** Vegetal fibres comprise psyllium and bamboo, inulin, and soya bran, among others. *** Gums additives include E412 (guar), E415 (xanthan). and E425 (konjac). **** Cellulose derivatives include additives E464 (hydroxypropyl methylcellulose), E460 (cellulose), and E461 (methylcellulose).

**Table 6:** Types of fibres used in the formulation of gluten-free breads.

	Food Subgroups (n)	Wheat Bran n (%)	Cereal Fibres * n (%)	Vegetal Fibres ** n (%)	Gums *** n (%)	Cellulose Derivatives **** n (%)	Fibre Additives (Total) n (%)	Fibre Ingredients (Total) n (%)
**Gluten-free breads**	Loaf (2)	0	0	1 (50.0)	2 (100.0)	2 (100.0)	2 (100)	1 (50.0)
	Baguette (3)	0	0	3 (100.0)	2 (66.7)	3 (100.0)	3 (100.0)	3 (100.0)
	Ciabatta (2)	0	0	2 (100.0)	0	2 (100.0)	2 (100)	2 (100.0)
	White sliced (3)	0	0	2 (66.7)	2 (66.7)	3 (100.0)	3 (100.0)	2 (66.7)
	Seeded sliced (3)	0	0	2 (66.7)	2 (66.7)	3 (100.0)	3 (100.0)	2 (66.7)
	Multigrain loaf (3)	0	0	3 (100.0)	0	1 (33.3)	3 (100.0)	3 (100.0)
	Muffin (3)	0	0	3 (100.0)	2 (66.7)	2 (66.7)	3 (100.0)	3 (100.0)
	Hamburger bun (3)	0	0	2 (66.7)	1 (33.3)	3 (100.0)	3 (100.0)	2 (66.7)
	Hot dog bun (2)	0	0	2 (100.0)	0	2 (100.0)	2 (100)	2 (100.0)
	**Total special bread (24)**	**0**	**0**	**20 (83.3)**	**11 (45.8)**	**21 (87.5)**	**24 (100.0)**	**20 (83.3)**
	Rusks (3)	0	0	2 (66.7)	1 (33.3)	2 (66.7)	3 (100.0)	2 (66.7)
	Breadsticks (3)	0	0	1 (33.3)	3 (100.0)	1 (33.3)	3 (100.0)	1 (33.3)
	Breadcrumbs (3)	0	0	1 (33.3)	3 (100.0)	2 (66.7)	3 (100.0)	2 (66.7)
	**Total special (dry) bread (9)**	**0**	**0**	**4 (44.4)**	**7 (77.8)**	**5 (55.6)**	**9 (100.0)**	**5 (55.6)**

Results are expressed as frequency (n: number of products including a specific ingredient) and (percentage based on the total products within the category or the subgroup). * Cereal fibres include wheat and oat fibre. ** Vegetal fibres comprise psyllium and bamboo, inulin, and soya bran, among others. *** Gums additives include E412 (guar), E415 (xanthan), and E425 (konjac). **** Cellulose derivatives include additives E464 (hydroxypropyl methylcellulose), E460 (cellulose), and E461 (methylcellulose).

**Table 7:** Moisture, fat, and fibre content in gluten-containing and gluten-free bread.

		Food Subgroups (n)	Moisture (g/100 g)	Fat (g/100 g)	Fibre (g/100 g)
**Gluten-containing breads**	**Common bread**	Loaf (3)	30.47 ± 0.14	1.64 ± 0.05	3.10 ± 0.49
		Baguette (3)	26.67 ± 0.04	1.13 ± 0.19	3.17 ± 0.20
		Ciabatta (3)	29.72 ± 0.09	1.34 ± 0.02	2.20 ± 0.15
		**Mean ± SD** **Median (IQR)**	**28.96 ± 1.75** **29.70 (26.69–30.39)**	**1.37 ± 0.24** **1.34 (1.24–1.62)**	**2.82 ± 0.54** **2.95 (2.28–3.28)**
	**Special bread**	White sliced (3)	36.13 ± 0.04	2.68 ± 0.02	3.06 ± 0.35
		Wholemeal sliced (3)	37.21 ± 0.15	3.00 ± 0.15	6.29 ± 0.84
		Rye sliced (3)	33.30 ± 0.16	5.82 ± 0.06	7.88 ± 0.56
		Seeded loaf (3)	34.04 ± 0.19	5.25 ± 0.01	5.26 ± 0.36
		Hamburger bun (3)	33.51 ± 0.13	4.66 ± 0.12	4.16 ± 1.38
		Hot dog bun (3)	33.33 ± 0.13	3.66 ± 0.06	2.31 ± 0.44
		**Mean ± SD** **Median (IQR)**	**34.59 ± 1.57** **33.77 (33.40–36.15)**	**4.18 ± 1.19** **4.15 (3.03–5.25)**	**4.86 ± 2.08** **5.13 (2.91–6.65)**
	**Special (dry) bread**	Rusks (3)	3.25 ± 0.03	6.07 ± 0.03	6.54 ± 0.94
		Breadsticks (3)	3.80 ± 0.05	6.15 ± 0.01	4.17 ± 1.67
		Breadcrumbs (3)	6.85 ± 0.03	2.11 ± 0.04	5.12 ± 0.67
		**Mean ± SD** **Median (IQR)**	**4.64 ± 1.68** **3.78 (3.27–6.84)**	**4.78 ± 2.00** **6.05 (2.13–6.15)**	**5.27 ± 1.45** **5.43 (4.62–6.06)**
**Gluten-free breads**	**Special bread**	Loaf (2)	35.25 ± 0.07	1.16 ± 0.09	2.61 ± 0.30
		Baguette (3)	38.98 ± 0.02	9.09 ± 0.09	9.31 ± 0.42
		Ciabatta (2)	30.33 ± 0.07	7.84 ± 0.09	8.64 ± 1.29
		White sliced (3)	38.80 ± 0.08	8.27 ± 0.06	8.17 ± 1.20
		Seeded sliced (3)	37.16 ± 0.11	7.52 ± 0.23	9.57 ± 0.97
		Multigrain loaf (3)	36.26 ± 0.35	7.98 ± 0.09	9.12 ± 1.22
		Muffin (3)	36.65 ± 0.25	4.88 ± 0.12	5.21 ± 0.46
		Hamburger bun (3)	38.83 ± 0.06	6.56 ± 0.09	7.15 ± 0.29
		Hot dog bun (3)	34.10 ± 0.12	6.34 ± 0.04	6.27 ± 1.59
		**Mean ± SD** **Median (IQR)**	36.26 ± 2.68 *† **36.71 (35.18–38.79)**	6.63 ± 2.30 *† **7.58 (6.31–8.07)**	7.34 ± 2.36 *† **7.44 (5.73–9.27)**
	**Special (dry) bread**	Rusks (3)	5.74 ± 0.03	7.88 ± 0.11	7.51 ± 0.77
		Breadsticks (3)	2.76 ± 0.05	7.64 ± 0.04	7.20 ± 1.76
		Breadcrumbs (3)	5.99 ± 0.05	6.23 ± 0.06	3.52 ± 1.62
		**Mean ± SD** **Median (IQR)**	**4.83 ± 1.55** **5.75 (2.79–5.96)**	**7.25 ± 0.78 #** **7.62 (6.25–7.85)**	**6.08 ± 2.29** **6.79 (4.40–8.15)**

Results are expressed in mean (g/100 g product) ± SD standard deviation and median (Percentile 25–Percentile 75). GC (n = 36) vs. GF (n = 36) breads (moisture *p* = 0.009; fat *p* < 0.001; fibre *p* < 0.001). * Common bread GC vs. Special bread GF (moisture *p* < 0.001; fat *p* < 0.001; fibre *p* < 0.001). † Special bread GC vs. Special bread GF (moisture *p* = 0.005; fat *p* < 0.001; fibre *p* = 0.002). # Special dry bread GC vs. Special dry bread GF (moisture *p* = 0.691; fat *p* < 0.001; fibre *p* = 0.354).

## Data Availability

The analytical data on GF and GC flours and breads will be made available in the institutional repository at Universidad San Pablo-CEU [CEU Repositorio Institucional] at (https://hdl.handle.net/10637/18475). Data will also be available on request from the corresponding author.

## Supplementary Materials

The following supporting information can be downloaded at https://www.mdpi.com/article/10.3390/foods14050894/s1, Table S1: List of ingredients of gluten-containing flours and ready-to-use flour mixes; Table S2: List of ingredients of gluten-free flours and ready-to-use flour mixes; Table S3: List of ingredients of gluten-containing breads; Table S4: List of ingredients of gluten-free breads; Table S5: Fat and fibre content of flour samples according to the label; Table S6: Fat and fibre content of bread samples according to their label; Table S7: Flour and starch sources used in the formulation of gluten-containing flour samples; Table S8: Flour and starch sources used in the formulation of gluten-free flour samples; Table S9: Flour sources used in the formulation of gluten-containing breads; Table S10: Starch and sourdough sources used in the formulation of gluten-containing breads; Table S11: Flour sources used in the formulation of gluten-free breads; Table S12: Starch and sourdough sources used in the formulation of gluten-free breads.
