# Supplementary material for: Determination and Comparison of Fat and Fibre Contents in Gluten-Free and Gluten-Containing Flours and Breads: Nutritional Implications
Source: Foods. 2025 Mar 5;14(5):894. doi: 10.3390/foods14050894 (PMC11899709; doi:10.3390/foods14050894)
Supplement: Supplementary file 1 [file foods-14-00894-s001.zip › foods-3412375-supplementary.pdf]

SUPPLEMENTARY TABLES

Table S1. List of ingredients of gluten-containing flours and ready-to-use flour mixes.

CATEGORY	#	INGREDIENTS
**Wheat**	1	Wheat flour (contains gluten).
	2	Wheat flour.
	3	Wheat flour.
**Whole wheat**	1	Wholemeal wheat flour.
	2	Wholemeal wheat flour (contains gluten)
	3	Wholemeal wheat flour.
**Wheat, strong**	1	Wheat flour.
	2	Strong wheat flour.
	3	Wheat flour (strong), flour treatment agent (ascorbic acid) and enzymes.
**Wholemeal spelt**	1	100% wholemeal spelt flour.
	2	Wholemeal spelt wheat flour.
	3	Wholemeal spelt (wheat) flour.
**Whole grain oat**	1	100% whole grain oat flour.
	2	Whole grain oat flour.
	3	100% whole grain oat flour.
**Brown rice**	1	100% organic brown rice, no additives.
	2	Flour produced by milling brown rice (100%).
	3	Rice flour.
**Maize**	1	Maize flour.
	2	Maize flour.
	3	Corn flour (100%) from organic agriculture.
**Wholemeal rye**	1	Wholemeal rye flour.
	2	Wholemeal rye flour.
	3	Wholemeal rye flour (gluten).
**Multi-grain**	1	Wheat (*), rye (*), barley (*), rice (*), oats (*). (*)=from organic agriculture.
	2	Wheat flour 65%, wholemeal rye flour 11%, wholemeal spelt flour 11%, oat flour 11%, wheat gluten, enzymes.
	3	Organic soft wheat flour (90%), organic wholemeal multigrain flour (wheat, rye, barley, rice, oats) (10%).
**Baking mix**	1	Wheat flour (contains gluten).
	2	Wheat flour.
	3	Wheat flour (gluten).
**Cake mix**	1	98% wheat flour, raising agent: sodium carbonates.
	2	Wheat flour, raising agents (3%): disodium diphosphate, sodium hydrogen carbonate.
	3	Wheat flour, raising agents (E-450i, E-500ii; anti-caking agent: E-170i; acidity regulator: E-516; maize starch).
**Pancake and crepe mix**	1	Wheat flour (contains gluten), skimmed milk powder, sugar, pasteurised egg powder, raising agent: sodium hydrogen carbonate.
	2	Wheat flour, skimmed milk powder, sugar, dried egg powder, raising agent 2% (sodium bicarbonate).
	3	Wheat flour, sugar, egg powder, milk powder, whey, raising agent (E-450i, E-500ii, anti-caking agent: E-170 i; acidity regulator: E-516; maize starch).
**Frying and battering mix**	1	Wheat flour (contains gluten) (80%), wheat semolina (contains gluten) (20%).
	2	Wheat flour (80%), wheat semolina (20%).
	3	Soft wheat semolina, durum wheat flour
**Pizza mix**	1	Wheat flour (90%) (contains gluten), yeast, raising agent: sodium carbonates.
	2	Wheat flour, yeast (8%) and raising agent (bicarbonate of soda)

	3	Wheat flour, toasted wheat germ and baking powder.
**Bread mix**	1	76% wheat flour, 20% rye flour, salt, dehydrated yeast, barley malt, flour treatment agent: ascorbic acid; emulsifier: sorbitan monostearate.
	2	Strong wheat flour, rye flour, wheat sourdough powder (3%), salt, dry baking powder and roasted barley malt flour.
	3	Wheat flour, millet, brown linseed, rye flour, sunflower seeds, wheat gluten, wheat semolina, sourdough, rice flour, salt, flour treatment agent (ascorbic acid) and enzymes.

# indicates the brand, alphabetically ordered among the group.

Table S2. List of ingredients of gluten-free flours and ready-to-use flour mixes

CATEGORY	#	INGREDIENTS
**Maize**	1	Organic maize grain.
	2	100% maize flour.
	3	Maize flour.
**Rice**	1	100% rice flour.
	2	Rice flour
	3	Rice flour.
**Brown rice**	1	Gluten-free brown rice flour* (*) from controlled organic agriculture.
	2	100% brown rice flour
	3	100% organically grown brown rice flour
**Whole grain oat**	1	Whole grain oat flour, gluten-free.
	2	Oat flour from organic farming.
	3	Gluten-free whole grain oat flour* (*) from organic agriculture.
**Teff**	1	Raw teff flour.
	2	Wholemeal teff flour.
	3	Teff flour.
**Millet**	1	Millet flour.
	2	Flour produced by milling millet.
	3	Flour produced by milling millet.
**Sorghum**	1	Sorghum flour.
	2	Flour produced from milling sorghum.
	3	Flour produced from milling sorghum.
**Wholemeal buckwheat**	1	Wholemeal buckwheat flour, gluten-free.
	2	Organically grown buckwheat flour.
	3	100% buckwheat flour from organic agriculture.
**Quinoa**	1	Quinoa flour.
	2	Quinoa flour.
	3	Flour from milling quinoa.
**Amaranth**	1	Amaranth flour from organic production.
	2	100% amaranth flour*. (*) organically grown
	3	Flour from grinding amaranth
**Chickpea**	1	Chickpea flour.
	2	100% chickpea flour.
	3	Chickpea flour.
**Potato starch**	1	Native potato starch.
	2	100% potato starch from organic production.
	3	Potato starch.
**Baking mix**	1	Maize starch, maize flour, rice flour, tapioca starch, soy flour, egg albumin, emulsifier (mono- and diglycerides of fatty acids), thickener (xanthan gum).
	2	Rice flour, maize starch, raising agents (sodium hydrogen carbonate, citric acid) and stabiliser (xanthan gum).
	3	Maize starch, maize flour, rice flour, lentil flour, modified tapioca starch, thickener: gum tara.

**Bread mix**	1	Maize starch, rice flour, buckwheat flour, dextrose, vegetable fibre (psyllium), salt, thickeners (hydroxypropyl methylcellulose)
	2	Rice flour, maize starch, dextrose, vegetable fibres (psyllium and bamboo), pea protein and stabiliser (hydroxypropyl methylcellulose).
	3	Maize starch, rice flour, vegetable fibres (psyllium, bamboo), brown rice flour 3.8%, lentil flour 3.6%, dextrose, thickener: hydroxypropyl methylcellulose; salt.
**Multipurpose mix**	1	Maize flour, maize starch, pea protein and thickener (xanthan gum).
	2	Maize starch, rice flour, potato flour, modified potato starch, sugar, thickeners (E412, E464, E401, E440), raising agents (E575, E500), iron, vitamins (thiamine, riboflavin, niacin, B6, folic acid).
	3	Corn starch, sugar, thickener: xanthan gum, emulsifier: E472e, salt, raising agents: E450, E500.

# indicates the brand, alphabetically ordered among the group.

Table S3. List of ingredients of gluten-containing breads.

CATEGORY	#	INGREDIENTS
**Loaf**	1	Wheat flour, water and yeast.
	2	Wheat flour (gluten), water, yeast, salt, emulsifier E 472e, antioxidant E 300.
	3	Wheat flour, water, yeast, salt and emulsifier (sunflower lecithin).
**Baguette**	1	Wheat flour, water, yeast, salt, inactive rye sourdough, emulsifier: E-472e and flour treatment agent: E-300
	2	Wheat flour (gluten), water, wheat sourdough (3%) (gluten), yeast, salt, antioxidant E 300.
	3	Wheat flour, water, yeast, salt and emulsifier (sunflower lecithin).
**Ciabatta**	1	Wheat flour, water, salt, dehydrated inactive wheat sourdough, yeast, antioxidant E300 and enzymes.
	2	Wheat flour (gluten), water, rye flour (1.9%) (gluten), salt, extra-fine wheat bran (gluten), yeast.
	3	Wheat flour, water, salt, yeast, wheat and rye sourdough and flour treatment agent (ascorbic acid).
**White sliced**	1	Wheat flour, water, yeast, vegetable oil (refined olive oil 1.4%), salt, inactive wheat and whole rye sourdough.
	2	Wheat flour, water, sunflower oil (3%), yeast, sugar, salt, bean flour, emulsifiers (sodium stearoyl-2-lactylate and fatty acid monoglycerides and diglycerides), wheat gluten, preservatives (calcium propionate and sorbic acid), stabiliser (guar gum), flour treatment agent: ascorbic acid and flavouring.
	3	Wheat flour (gluten), water, yeast, sugar, sunflower oil (1%), salt, wine vinegar, bean flour, wheat gluten, wheat fibre (gluten), emulsifiers (E-472e, E-471), preservatives (E-282, E-202, E-200), flour treatment agent (E-300)
**Wholemeal sliced**	1	Whole wheat flour, water, yeast, sugar, wheat gluten, vegetable oil (refined olive 1.8%), salt, inactive whole rye sourdough, inactive maize sourdough.
	2	Whole wheat flour (gluten) (59%), water, yeast, wheat gluten, sugar, sunflower oil (1%), salt, wheat fibre (gluten), wine vinegar, whole malted barley flour (gluten) (0.2%), emulsifiers (E-472e, E-471), preservatives (E-282, E-202, E-200), antioxidant (E-200).
	3	Whole wheat flour (54%), water, yeast, olive oil, wheat gluten, vegetable fibre, whole malted barley flour (0.9%), salt, vinegar, emulsifiers: E471, E472e (contains wheat), preservatives (E200, E282), flavouring, flour treatment agent: ascorbic acid (contains wheat).
**Rye sliced**	1	Rye flour (28%), wheat flour, water, yeast, sesame seeds (3.5%), poppy seeds (3&), roasted malt flour (barley), sugar, sunflower oil (1.3%), inactive wheat sourdough, salt, wine vinegar and preservative (sorbic acid).
	2	Rye flour (gluten) 22% and wheat flour (gluten), water, wheat bran (gluten), wheat gluten, yeast, roasted malted barley flour (gluten), glucose and fructose syrup, sunflower oil, salt, vinegar and preservatives (calcium propionate and ascorbic acid).
	3	Rye flour (26.7%), whole wheat flour, water, sunflower seeds (11%), wheat gluten, malted whole barley flour (3.2%), sugar, yeast, vegetable oil (sunflower), salt, preservatives (E282, E200), wine vinegar, emulsifier (E481), wheat flour, flour treatment agents (E300, E341).
**Seeded loaf**	1	Wheat flour, water, chia seeds (3%), sesame seeds (2.5%), flax seeds (2.5%), yeast, inactive rye sourdough, sunflower oil, inactive wheat sourdough, salt and wine vinegar.

CATEGORY	#	INGREDIENTS
	2	Wheat flour (gluten), water, salt, sunflower seeds (3.3%), sesame (2.6%), wheat bran (gluten), millet (2.1%), linseed (2.1%), rye flour (gluten) (1%), sunflower oil, yeast, wheat gluten, bread improver [wheat flour (gluten) and flavouring], preservative E-282, guar gum, rice flour, maize starch.
	3	Wheat flour, water, active wheat sourdough (12%), linseed (4.7%), rye flour (4%), yeast, vegetable oil (sunflower), inactive wheat sourdough, rye flakes, salt, wheat fibre, chopped spelt wheat (0, 8%), oat flakes (0.8%), potato flakes, chopped pumpkin seeds, wheat gluten, chopped barley, wheat germ, maize flour, sunflower seeds, sesame seeds, chopped buckwheat, wheat semolina.
**Hamburger bun**	1	Wheat flour, water, yeast, sugar, sesame seeds (2%), vegetable oil (sunflower), salt, wheat gluten, emulsifiers (E471, E481, E472e), preservatives (E282, E200, E202), broad bean flour, wine vinegar, acidity regulator (E341), flour treatment agent (E300).
	2	Wheat flour, water, yeast, sugar, sesame seeds (2%), vegetable oil (sunflower) (1.8%), salt, wheat gluten, emulsifiers (fatty acid monoglycerides and diglycerides, sodium steroyl-2-lactylate, monoacetyl and diacetyl tartaric esters of monoglycerides and diglycerides of fatty acids), preservatives (calcium propionate, sorbic acid, potassium sorbate), bean flour, wine vinegar, acidity regulator (calcium phosphates), flour treatment agent (ascorbic acid)
	3	Wheat flour, water, yeast, sugar, sunflower oil, sesame seeds, salt, wheat gluten, emulsifiers (E471, E481), preservatives (E282, E200, E202), stabiliser (guar gum), acidity regulator (E262), flour treatment agent (ascorbic acid).
**Hot dog bun**	1	Wheat flour, water, yeast, sugar, vegetable oil (sunflower), salt, wheat gluten, preservatives (E282, E200, E202), wine vinegar, emulsifiers (E471, E481, E472e), bean flour, acidity regulator (E341), flour treatment agent (E300).
	2	Wheat flour, water, yeast, sunflower oil (3%), sugar, oat fibre, salt, wheat gluten, emulsifiers: mono- and diglycerides of fatty acids and sodium stearoyl 2-lactylate; stabiliser: guar gum, preservatives: sorbic acid, calcium propionate and potassium sorbate; flavourings and acidity regulator: sodium acetate.
	3	Wheat flour (gluten), water, yeast, sugar, sunflower vegetable oil (2%), salt, wheat fibre (gluten), wine vinegar, broad bean flour, wheat gluten, emulsifiers (E472e, E471), preservatives (E282, E202), flour treatment agent (E300).
**Rusks**	1	Wheat flour 86.3%, sunflower oil, sugar, salt, yeast, wheat gluten.
	2	Wheat flour, water, high oleic sunflower oil (3.1%), sugar, salt, yeast, wheat gluten, emulsifiers (E481 and E472e) and antioxidant (E300).
	3	Wheat flour 90%, yeast, sugar, oleic sunflower oil 1.9%, salt, olive oil 1.1%, whey (milk), malted barley flour, wheat gluten, flour treatment agent: ascorbic acid
**Breadsticks**	1	Wheat flour (69.5%) (contains gluten), water, salt, yeast, and emulsifier (E472e).
	2	Wheat flour, water, salt, yeast, extra virgin olive oil (1%)
	3	Wheat flour, refined high oleic sunflower oil, yeast and sea salt.
**Breadcrumbs**	1	Wheat flour, water, salt and yeast.
	2	Wheat flour, yeast and salt.
	3	Wheat flour, yeast, salt.

# indicates the brand, alphabetically ordered among the group.

Table S4. List of ingredients of gluten-free breads.

CATEGORY	#	INGREDIENTS
**Loaf**	1	Water, maize starch, rice flour, yeast, maize flour, sugar, salt, stabiliser: E412 and E461 and raising agent: E500ii
	2	Maize starch, rice flour, water, yeast, invert sugar syrup, sugar, salt, olive oil, stabilisers (guar gum, hydroxypropyl methylcellulose, calcium diphosphate), psyllium fibre, raising agent (sodium bicarbonate).
**Baguette**	1	Water, corn starch, high oleic sunflower oil (6%), glucose and fructose syrup, vegetable fibres (psyllium and bamboo), whole grain rice flour, dextrose, quinoa and buckwheat sourdough, yeast, tapioca starch, egg albumin** powder, salt, stabilisers (hydroxypropyl methylcellulose and xanthan gum), acidity regulator (monocalcium phosphate), raising agent (sodium hydrogen

CATEGORY	#	INGREDIENTS
		carbonate), preservatives (calcium propionate and potassium sorbate), emulsifiers (monoglycerides and diglycerides of fatty acids and antioxidants (ascorbic acid).
	2	Water, maize starch, high oleic sunflower oil (8%), vegetable fibres (psyllium and bamboo), yeast, rice flour, rice syrup, sugar, inactive sourdough, tapioca starch, pea protein, stabilisers (hydroxypropyl methylcellulose and xanthan gum), salt, acidity regulator (monocalcium phosphate), raising agent (sodium hydrogen carbonate), flavourings, preservatives (potassium sorbate, calcium propionate), emulsifiers (monoglycerides and diglycerides of fatty acids) and antioxidant (ascorbic acid).
	3	Maize starch, water, sourdough 19% (rice flour, water), rice flour, vegetable fibres (psyllium, bamboo), high oleic sunflower oil, soy protein, thickener: hydroxypropyl methylcellulose, rice starch, maize germ, yeast, rice syrup, iodised salt (salt, potassium iodide).
**Ciabatta**	1	Water, rice flour, rice starch, tapioca starch, sugar, yeast, sunflower oil, inactive sourdough, thickener: E464, psyllium fibre, flavourings, rapeseed oil, emulsifier: E471, salt, preservatives: E202, E281.
	2	Maize starch, sourdough 14% (rice flour, water), water, rice flour, sunflower seeds 7%, buckwheat flour 5.5%, linseed 5%, yeast, vegetable fibres (psyllium, bamboo), apple extract, rice starch, thickener: hydroxypropyl methylcellulose, sunflower oil, rice syrup, integral rice flour, soy protein, millet flour, extra virgin olive oil, sea salt, acidulants: citric acid, tartaric acid.
**White sliced**	1	Water, maize starch, sourdough (rice flour, water), tapioca starch, buckwheat flour, sunflower oil, rice syrup, vegetable fibres (psyllium, bamboo), quinoa flour, rice protein, yeast, sugar, salt, thickener (hydroxypropyl methylcellulose), emulsifier (mono- and diglycerides of fatty acids), flavouring, preservatives (sorbic acid, calcium propionate).
	2	Maize starch, water, sourdough 10% (water, rice flour, buckwheat flour, quinoa flour, yeast), sunflower seed oil, sugar, soy proteins, psyllium fibre, thickeners: hydroxypropyl methylcellulose, guar gum; yeast, emulsifier: monoglycerides and diglycerides of fatty acids; salt, yeast extract, flavourings.
	3	Water, corn starch, tapioca starch, high oleic sunflower oil (7%), potato starch, sugar, yeast, thickeners: xanthan gum, E464; salt, egg, emulsifiers: E471, E481; raising agents: E450, E500; preservative: E281, antioxidant: ascorbic acid.
**Seeded sliced**	1	Water, maize starch, tapioca starch, rice flour, potato starch, vegetable oil (sunflower), yeast, inulin, egg white powder, psyllium fibre, thickeners (E415, E464), linseed (1.7%), sunflower seeds (1.1%), sugar, carob flour, poppy seeds (0.4%), salt, preservative (E281), antioxidant (E300), flavourings.
	2	Water, maize starch, tapioca starch, sunflower oil (6%), potato starch, sugar, yeast, thickeners (xanthan gum, hydroxypropyl methylcellulose), linseed (1.8%), invert sugar syrup, sunflower seeds (1.2%), egg, salt, poppy seeds (0,4%), emulsifiers (monoglycerides and diglycerides of fatty acids, sodium stearoyl-2-lactylate), carob flour, raising agents (diphosphates, sodium carbonates), preservative (sodium propionate), antioxidant (ascorbic acid), antioxidant (ascorbic acid).
	3	Maize starch, water, sourdough 14% (rice flour, water), rice starch, vegetable fibre (psyllium), rice syrup, sunflower oil, soy flakes, sunflower seeds 2.1%, millet flour 2%, linseed 1.9%, thickener: hydroxypropyl methylcellulose; quinoa flour 1.3%, yeast, rice flour 1.3%, soy protein, soy bran, iodised salt (salt, potassium iodide), poppy seeds 0.6%, chia seeds (*Salvia hispanica*) 0.6%, honey.
**Multigrain loaf**	1	Water, maize starch, sourdough (rice flour 7.5%, water), tapioca starch, seeds 8.9% (flax 4.5%, sunflower 2.2%, millet 2.2%), sunflower oil, buckwheat flour 3.6%, rice syrup, millet flour 1.8%, vegetable fibre (psyllium), flax flour 0.36%, carob flour 0.25%, rice protein, sugar, yeast, salt, thickener (hydroxypropyl methylcellulose), emulsifier (mono- and diglycerides of fatty acids), preservatives (sorbic acid, calcium propionate).
	2	Water, maize starch, sourdough (rice flour 7.5%, water), tapioca starch, seeds 8.9% (flax 4.5%, sunflower 2.2%, millet 2.2%), sunflower oil, buckwheat flour 3.6%, rice syrup, millet flour 1.8%, vegetable fibre (psyllium), flax flour 0.36%, carob flour 0.25%, rice protein, sugar, yeast, salt, thickener (hydroxypropyl methylcellulose), emulsifier (monoglycerides and diglycerides of fatty acids), preservatives (sorbic acid, calcium propionate).

CATEGORY	#	INGREDIENTS
	3	Maize starch, water, sourdough 12% (rice flour, water), buckwheat flour 4%, rice flour, flaxseed 3.7%, vegetable fibre (psyllium), rice starch, sugar syrup, sunflower seeds 3.2%, thickeners: hydroxypropyl methylcellulose, cellulose; yeast, soy protein, sea salt, sunflower oil, millet flour 1.5%, brown rice flour 1.5%, chia seeds (*Salvia hispanica*) 1.3%, apple extract, sugar.
**Muffin**	1	Water, maize starch, rice flour, buckwheat flour, sunflower seed oil, psyllium fibre, rice starch, emulsifier: monoglycerides and diglycerides of fatty acids; extra virgin olive oil, glucose-fructose syrup, salt, yeast, sugar, thickener: guar gum, xanthan gum; yeast extract, flavourings, potato protein.
	2	Maize starch, rice flour, water, yeast, invert sugar syrup, sugar, salt, olive oil (1.1%), stabilisers (guar gum, hydroxypropyl methylcellulose, calcium diphosphate), psyllium fibre, raising agent (sodium bicarbonate).
	3	Maize starch, water, sourdough 14% (rice flour, water), vegetable fibre (psyllium), millet flour, sunflower oil, rice flour, soy protein, rice starch, thickener: hydroxypropyl methylcellulose; rice syrup, salt, yeast, sugar.
**Hamburger bun**	1	Maize starch, water, cream margarine (palm vegetable fat, water, sunflower vegetable oil, emulsifier (mono and diglycerides of fatty acids), salt, acidifier (citric acid) and antioxidants (tocopherol-rich extract, ascorbyl palmitate)), potato starch, maize flour, thickener (xanthan gum), sugar, yeast, salt, raising agent (sodium bicarbonate), preservative (sodium propionate), thickener (hydroxypropyl methylcellulose), antioxidant (vitamin C), wine vinegar, preservative (sorbic acid) and thickener (konjac gum).
	2	Maize starch, water, sugar, sunflower seed oil, rice flour, thickener: hydroxypropyl methylcellulose; yeast, emulsifier: monoglycerides and diglycerides of fatty acids; psyllium fibre, soy proteins, salt, raising agent: disodium diphosphate, sodium hydrogen carbonate; flavourings.
	3	Sourdough 25% (rice flour, water), water, rice starch, maize starch, millet flour, vegetable fibre (psyllium), sugar, thickener: hydroxypropyl methylcellulose; lupin protein, sunflower oil, yeast, salt, emulsifier: monoglycerides and diglycerides of fatty acids, flavouring.
**Hot dog bun**	1	Maize starch, water, sugar, sunflower seed oil, rice flour, thickener: hydroxypropyl methylcellulose; yeast, emulsifier: mono- and diglycerides of fatty acids; psyllium fibre, soy proteins, salt, raising agent: disodium diphosphate, calcium carbonate; flavours
	2	Maize starch, sourdough 19% (rice flour, water), rice flour, vegetable fibres (psyllium, bamboo), high oleic sunflower oil, soy protein, thickener: hydroxypropyl methylcellulose; rice starch, maize germ, yeast, rice syrup, iodised salt (salt, potassium iodide).
**Rusks**	1	Potato starch, maize starch, rice flour, sunflower oil, sugar, thickener: E464, psyllium fibre, yeast, salt, potato protein, emulsifier: monoglycerides and diglycerides of fatty acids, citrus fibres, antioxidant: tocopherol-rich extract, flavourings.
	2	Corn starch, water, sugar, egg, sunflower oil, thickener: xanthan gum, yeast, salt, emulsifier: E472e, raising agents: E450, E500; antioxidant: ascorbic acid
	3	Maize starch, rice flour, high oleic sunflower oil, sugar, vegetable fibres (psyllium, bamboo), rice syrup, thickener: hydroxypropyl methylcellulose; buckwheat flour, lentil flour, pea proteins, yeast, salt, natural flavouring
**Breadsticks**	1	Maize starch, water, rice flour, extra virgin olive oil 3%, vegetable fibres, yeast, sea salt, sugar, thickeners (guar gum, hydroxypropyl methylcellulose), raising agents (disodium diphosphate, sodium carbonates).
	2	Maize starch, buckwheat flour 28%, rice flour, vegetable fat (coconut), yeast, vegetable oil (sunflower) 2%, thickener: guar gum, salt, antioxidant: rosemary extract.
	3	Maize starch, egg powder, wholemeal rice flour, potato starch, dextrose, high oleic sunflower oil (13%), stabiliser: xanthan gum, emulsifiers: monoacetyl and diacetyl tartaric esters of monoglycerides and diglycerides of fatty acids, salt, acidity regulator: monocalcium phosphate, antioxidant: ascorbic acid, natural flavourings.
**Breadcrumbs**	1	Maize starch, water, rice flour, tapioca starch, yeast, dextrose, sugar, extra virgin olive oil (2%), salt, thickeners (guar gum, E464), emulsifier (monoglycerides and diglycerides of fatty acids), psyllium fibre, lupin protein, buckwheat flour.

CATEGORY	#	INGREDIENTS
	2	Maize starch, maize flour, salt, stabiliser (guar gum, hydroxypropyl methylcellulose, xanthan gum), raising agent (sodium hydrogen carbonate), sugar, preservative (potassium sorbate), water and yeast.
	3	Rice flour, maize starch, soy flour, palm fat, egg, glucose syrup, thickener: guar seed flour; yeast, salt.

# indicates the brand, alphabetically ordered among the group.

Table S5. Fat and fibre content of flour samples according to the label.

Cereal or cereal-like milling products and derivatives	Food subgroups		Fat (%)	Fibre (%)
**Gluten-containing products**	**Flours**	Wheat	1.93 ± 0.70	4.00 ± 1.41
		Whole wheat	2.10 ± 0.00	11.13 ± 2.66
		Wheat, strong	1.40 ± 0.21	2.30 ± 0.28
		Wholemeal spelt	2.05 ± 0.25	8.76 ± 1.08
		Whole grain oat	7.40 ± 1.87	14.23 ± 1.08
		Brown rice	1.70 ± 1.21	2.00 ± 1.41
		Maize	2.30 ± 0.99	2.60
		Wholemeal rye	1.83 ± 0.49	19.47 ± 2.34
		Multi-grain	2.00 ± 0.60	4.83 ± 2.11
	**Ready-to-use flour mixes**	Baking mix	1.73 ± 0.46	2.10
		Cake mix	1.40 ± 0.46	2.30 ± 0.99
		Pancake and crepe mix	4.40 ± 1.06	2.00 ± 1.41
		Frying and battering mix	1.50 ± 0.89	2.55 ± 0.64
		Pizza mix	1.47 ± 0.55	3.70 ± 0.99
		Bread mix	2.70 ± 2.44	4.20 ± 1.66
**Gluten-free products**	**Flours**	Maize	2.87 ± 1.15	8.47 ± 0.64
		Rice	1.45 ± 0.18	2.78
		Brown rice	2.13 ± 0.90	3.37 ± 1.07
		Whole grain oat	6.47 ± 0.40	10.37 ± 1.15
		Teff	1.90 ± 1.11	7.53 ± 064
		Millet	3.23 ± 0.31	2.70 ± 0.60
		Sorghum	2.07 ± 1.57	5.73 ± 1.25
		Wholemeal buckwheat	2.83 ± 0.51	4.10 ± 0.78
		Quinoa	6.68 ± 0.70	3.91 ± 4.04
		Amaranth	7.33 ± 1.33	8.37 ± 6.52
		Chickpea	5.67 ± 0.95	6.65 ± 4.74
	**Starch**	Potato	0.17 ± 0.15	0.50 ± 0.71
	**Ready-to-use flour mixes**	Baking mix	0.67 ± 0.57	1.75 ± 1.06
		Bread mix	0.70 ± 0.14	3.00 ± 1.56
		Multipurpose mix	0.93 ± 0.44	2.87 ± 1.77

Results are expressed in mean (g/100g product) ± standard deviation.

Table S6. Fat and fibre content of bread samples according to the label.

**Food subgroups**		**Fat (%)**	**Fibre (%)**	
**Gluten-containing breads**	**Common bread**	Loaf	1.80 ± 0.95	2.47 ± 0.38
		Baguette	1.20 ± 0.26	2.75 ± 0.07
		Ciabatta	1.20 ± 0.26	3.10 ± 1.41
	**Special bread**	White sliced bread	3.17 ± 1.29	3.43 ± 0.67
		Wholemeal sliced	2.83 ± 0.31	6.73 ± 0.64
		Rye sliced	5.83 ± 2.86	8.60 ± 3.35
		Seeded loaf	4.83 ± 1.17	6.53 ± 1.56
		Hamburger bun	3.80 ± 0.87	3.47 ± 0.23
		Hot dog bun	4.23 ± 0.49	3.77 ± 1.25
		Rusks	6.13 ± 1.15	4.43 ± 0.23
	**Special (dry) bread**	Breadsticks	5.63 ± 7.25	3.50 ± 0.79
		Breadcrumbs	1.77 ± 0.71	4.40 ± 0.52
**Gluten-free breads**	**Special bread**	Loaf	1.70 ± 0.28	
		Baguette	7.63 ± 3.41	8.17 ± 0.86
		Ciabatta	7.40 ± 2.83	6.60 ± 4.24
		White sliced	7.13 ± 1.86	4.07 ± 0.64
		Seeded sliced	7.80 ± 1.66	8.57 ± 2.40
		Multigrain loaf	6.90 ± 1.39	7.43 ± 0.92
		Muffin	4.60 ± 2.98	6.00 ± 1.27
		Hamburger bun	5.49 ± 2.61	3.77 ± 1.70
		Hot dog bun	4.95 ± 1.77	4.95 ± 3.46
	**Special (dry) bread**	Rusks	8.43 ± 2.51	7.37 ± 4.05
		Breadsticks	9.23 ± 4.75	5.05 ± 1.91
		Breadcrumbs	5.90 ± 7.04	3.27 ± 3.37

Results are expressed in mean (g/100g product) ± standard deviation.

Table S7. Flour and starch sources used in the formulation of gluten-containing flour samples.

Cereal or cereal-like milling products and derivatives	Food subgroups (n)	Wheat flour	Rye flour	Malted barley flour	Oat flour	Rice flour	Corn starch	Presence of sourdough	Wheat semolina	Presence of wholemeal flour
		n (%)	n (%)	n (%)	n (%)	n (%)	n (%)	n (%)	n (%)	n (%)
	**Flours (3)**	**3 (100.0)**	**1 (33.3)**	**0**	**2 (66.7)**	**1 (33.3)**	**0**	**0**	**0**	**2 (66.7)**
	Multi-grain (3)	3 (100.0)	1 (33.3)	0	2 (66.7)	1 (33.3)	0	0	0	2 (66.7)
	**Ready-to-use flour mixes (18)**	**3 (100.0)**	**3 (16.7)**	**3 (16.7)**	**0**	**0**	**2 (11.1)**	**2 (11.1)**	**3 (16.7)**	**0**
	Baking mix (3)	3 (100.0)	**0**	**0**	**0**	**0**	**0**	**0**	**0**	**0**
	Cake mix (3)	3 (100.0)	0	0	0	0	1 (33.3)	0	0	0
	Pancake and crepe mix (3)	3 (100.0)	0	0	0	0	1 (33.3)	0	0	0
	Frying and battering mix (3)	3 (100.0)	0	0	0	0	0	0	0	0
	Pizza mix (3)	3 (100.0)	0	0	0	0	0	0	3 (100.0)	0
	Bread mix (3)	3 (100.0)	3 (100.0)	3 (100.0)	0	0	0	2 (66.7)	0	0
	**Total (21)**	**21 (100.0)**	**4 (19.0)**	**3 (14.3)**	**2 (9.5)**	**1 (4.8)**	**2 (9.5)**	**2 (9.5)**	**3 (14.3)**	**2 (9.5)**

Results are expressed as frequency (n: number of products including a specific ingredient) and (percentage based on the total products within the category or the subgroup).

Table S8. Flour and starch sources used in the formulation of gluten-free flour samples.

Cereal or cereal-like milling products and derivatives	Food subgroups (n)	Maize flour	Rice flour	Wholemeal rice flour	Buckwheat flour	Potato flour	Legume flour	Maize starch	Tapioca starch	Potato starch
		n (%)	n (%)	n (%)	n (%)	n (%)	n (%)	n (%)	n (%)	n (%)
	**Ready-to-use flour mixes (9)**	**3 (33.3)**	**7 (77.8)**	**1 (11.1)**	**1 (11.1)**	**1 (11.1)**	**3 (33.3)**	**9 (100.0)**	**2 (22.2)**	**1 (11.1)**
	Baking mix (3)	2 (66.7)	3 (100.0)	0	0	0	2 (66.7)	3 (100.0)	2 (66.7)	0
	Bread mix (3)	0	3 (100.0)	1 (33.3)	1 (33.3)	0	1 (33.3)	3 (100.0)	0	0
	Multipurpose mix (3)	1 (33.3)	1 (33.3)	0	0	1 (33.3)	0	3 (100.0)	0	1 (33.3)
	**Total (9)**	**3 (33.3)**	**7 (77.8)**	**1 (11.1)**	**1 (11.1)**	**1 (11.1)**	**3 (33.3)**	**9 (100.0)**	**2 (22.2)**	**1 (11.1)**

Results are expressed as frequency (n: number of products including a specific ingredient) and (percentage based on the total products within the category or the subgroup).

Table S9. Flour sources used in the formulation of gluten-containing breads.

**Bread and similar**	**Food subgroups (n)**	**Wheat flour**	**Whole wheat flour**	**Rye flour**	**Malted barley flour**	**Malted wholemeal barley flour**	**Bean flour**	**Whole wheat flours**	**Cereal and tuber flakes**	**Cereal grains**
		**n (%)**	**n (%)**	**n (%)**	**n (%)**	**n (%)**	**n (%)**	**n (%)**	**n (%)**	**n (%)**
	**Common bread (9)**	**3 (100.0)**	**0**	**1 (11.1)**	**0**	**0**	**0**	**0**	**0**	**0**
	Loaf (3)	3 (100.0)	0	0	0	0	0	0	0	0
	Baguette (3)	3 (100.0)	0	0	0	0	0	0	0	0
	Ciabatta (3)	3 (100.0)	0	1 (33.3)	0	0	0	0	0	0
	**Special bread (18)**	**3 (100.0)**	**4 (22.2)**	**5 (27.8)**	**2 (11.1)**	**3 (16.7)**	**6 (33.3)**	**4 (22.2)**	**1 (5.6)**	**2 (11.1)**
	White sliced (3)	3 (100.0)	0	0	0	0	2 (66.7)	0	0	0
	Wholemeal (3)	0	3 (100.0)	0	0	2 (66.7)	0	3 (100.0)	0	0
	Rye sliced (3)	3 (100.0)	1 (33.3)	3 (100.0)	2 (66.7)	1 (33.3)	0	1 (33.3)	0	0
	Seeded loaf (3)	3 (100.0)	0	2 (66.7)	0	0	0	0	1 (33.3)	2 (66.7)
	Hamburger bun (3)	3 (100.0)	0	0	0	0	2 (66.7)	0	0	0
	Hot dog bun (3)	3 (100.0)	0	0	0	0	2 (66.7)	0	0	0
	**Special (dry) bread (9)**	**3 (100.0)**	**0**	**0**	**1 (11.1)**	**0**	**0**	**0**	**0**	**0**
	Rusks (3)	3 (100.0)	0	0	1 (33.3)	0	0	0	0	0
	Breadsticks (3)	3 (100.0)	0	0	0	0	0	0	0	0
	Breadcrumbs (3)	3 (100.0)	0	0	0	0	0	0	0	0
**Total (36)**		**33 (91.7)**	**4 (11.1)**	**6 (16.7)**	**3 (8.3)**	**3 (8.3)**	**6 (16.7)**	**4 (11.1)**	**1 (2.8)**	**2 (5.6)**

Results are expressed as frequency (n: number of products including a specific ingredient) and (percentage based on the total products within the category or the subgroup).

Table S10. Starch and sourdough sources used in the formulation of gluten-containing breads.

**Bread and similar**	**Food subgroups (n)**	**Wheat sourdough**	**Wheat and rye sourdough**	**Active sourdough**	**Inactivated wheat sourdough**	**Inactivated rye sourdough**	**Inactivated whole rye sourdough**	**Inactivated wholemeal wheat and rye sourdough**	**Inactivated maize sourdough**	**Inactive sourdough**	**Presence of sourdough**
		**n (%)**	**n (%)**	**n (%)**	**n (%)**	**n (%)**	**n (%)**	**n (%)**	**n (%)**	**n (%)**	**n (%)**
	**Common bread (9)**	**1 (11.1)**	**1 (11.1)**	**2 (22.2)**	**1 (11.1)**	**1 (11.1)**	**0**	**0**	**0**	**2 (11.1)**	**4 (44.4)**
	Loaf (3)	0	0	0	0	0	0	0	0	0	0
	Baguette (3)	1 (33.3)	0	1 (33.3)	0	1 (33.3)	0	0	0	1 (33.3)	2 (66.7)
	Ciabatta (3)	0	1 (33.3)	1 (33.3)	1 (33.3)	0	0	0	0	1 (33.3)	2 (66.7)
	**Special bread (18)**	**1 (5.6)**	**0**	**1 (5.6)**	**3 (16.7)**	**1 (5.6)**	**1 (5.6)**	**1 (5.6)**	**1 (5.6)**	**5 (27.8)**	**5 (27.8)**
	White sliced (3)	0	0	0	0	0	0	1 (33.3)	0	1 (33.3)	1 (33.3)
	Wholemeal sliced (3)	0	0	0	0	0	1 (33.3)	0	1 (33.3)	1 (33.3)	1 (33.3)
	Rye sliced (3)	0	0	0	1 (33.3)	0	0	0	0	1 (33.3)	1 (33.3)
	Seeded loaf (3)	1 (33.3)	0	1 (33.3)	2 (66.7)	1 (33.3)	0	0	0	2 (66.7)	2 (66.7)
	Hamburger bun (3)	0	0	0	0	0	0	0	0	0	0
	Hot dog bun (3)	0	0	0	0	0	0	0	0	0	0
	**Special (dry) bread (9)**	**0**	**0**	**0**	**0**	**0**	**0**	**0**	**0**	**0**	**0**
	Rusks (3)	0	0	0	0	0	0	0	0	0	0
	Breadsticks (3)	0	0	0	0	0	0	0	0	0	0
	Breadcrumbs (3)	0	0	0	0	0	0	0	0	0	0
**Total (36)**		**2 (5.6)**	**1 (2.8)**	**3 (8.3)**	**4 (11.1)**	**2 (5.6)**	**1 (2.8)**	**1 (2.8)**	**1 (2.8)**	**17 (19.4)**	**9 (25.0)**

Results are expressed as frequency (n: number of products including a specific ingredient) and (percentage based on the total products within the category or the subgroup).

Table S11. Flour sources used in the formulation of gluten-free breads.

Bread and similar	Food subgroups (n)	Maize flour n (%)	Rice flour n (%)	Wholemeal rice flour n (%)	Buckwheat flour n (%)	Millet flour n (%)	Quinoa flour n (%)	Linseed flour n (%)	Legume flour n (%)	Soybean flakes n (%)
	**Special bread (24)**	**3 (12.5)**	**12 (50.0)**	**3 (12.5)**	**6 (25.0)**	**5 (20.8)**	**2 (8.3)**	**2 (8.3)**	**2 (8.3)**	**1 (4.2)**
	Loaf (2)	1 (50.0)	2 (100.0)	0	0	0	0	0	0	0
	Baguette (3)	0	2 (66.7)	1 (33.3)	0	0	0	0	0	0
	Ciabatta (2)	0	2 (100.0)	1 (50.0)	1 (50.0)	0	0	0	0	0
	White sliced (3)	0	0	0	1 (33.3)	0	1 (33.3)	1 (33.3)	0	0
	Seeded sliced (3)	0	1 (33.3)	0	0	1 (33.3)	1 (33.3)	1 (33.3)	0	1 (33.3)
	Multigrain loaf (3)	0	1 (33.3)	1 (33.3)	3 (100.0)	2 (66.7)	0	0	2 (66.7)	0
	Muffin (3)	0	2 (66.7)	0	1 (33.3)	1 (33.3)	0	0	0	0
	Hamburger bun (3)	2 (66.7)	0	0	0	1 (33.3)	0	0	0	0
	Hot dog bun (2)	0	2 (100.0)	0	0	0	0	0	0	0
	**Special (dry) bread (9)**	**1 (11.1)**	**6 (6.67)**	**1 (11.1)**	**3 (33.3)**	**0**	**0**	**0**	**2 (22.2)**	**0**
	Rusks (3)	0	2 (66.7)	0	1 (33.3)	0	0	0	1 (33.3)	0
	Breadsticks (3)	0	2 (66.7)	1 (33.3)	1 (33.3)	0	0	0	0	0
	Breadcrumbs (3)	1 (33.3)	2 (66.7)	0	1 (33.3)	0	0	0	1 (33.3)	0
**Total (33)**		**4 (12.1)**	**18 (54.5)**	**4 (12.1)**	**9 (27.3)**	**5 (15.2)**	**2 (6.1)**	**2 (6.1)**	**4 (12.1)**	**1 (3.0)**

Results are expressed as frequency (n: number of products including a specific ingredient) and (percentage based on the total products within the category or the subgroup).

Table S12. Starch and sourdough sources used in the formulation of gluten-free breads.

Bread and similar	Food subgroups (n)	Maize starch	Rice starch	Tapioca starch	Potato starch	Rice sourdough	Rice, buckwheat and quinoa sourdough	Buckwheat and quinoa sourdough	Inactive sourdough	Presence of sourdough
		n (%)	n (%)	n (%)	n (%)	n (%)	n (%)	n (%)	n (%)	n (%)
	**Special bread (24)**	**23 (95.8)**	**10 (41.7)**	**9 (37.5)**	**4 (23.5)**	**10 (41.7)**	**1 (4.2)**	**1 (4.2)**	**2 (8.3)**	**14 (58.3)**
	Loaf (2)	2 (100.0)	0	0	0	0	0	0	0	0
	Baguette (3)	3 (100.0)	1 (33.3)	2 (66.7)	0	1 (33.3)	0	1 (33.3)	1 (33.3)	3 (100.0)
	Ciabatta (2)	1 (50.0)	2 (100.0)	1 (50.0)	0	1 (50.0)	1 (33.3)	0	1 (50.0)	2 (100.0)
	White sliced (3)	3 (100.0)	0	2 (66.7)	1 (33.3)	1 (33.3)	0	0	0	2 (66.7)
	Seeded sliced (3)	3 (100.0)	1 (33.3)	2 (66.7)	2 (66.7)	1 (33.3)	0	0	0	1 (33.3)
	Multigrain loaf (3)	3 (100.0)	1 (33.3)	2 (66.7)	0	3 (100.0)	0	0	0	3 (100.0)
	Muffin (3)	3 (100.0)	3 (100.0)	0	0	1 (33.3)	0	0	0	1 (33.3)
	Hamburger bun (3)	3 (100.0)	1 (33.3)	0	1 (33.3)	1 (33.3)	0	0	0	1 (33.3)
	Hot dog bun (2)	2 (100.0)	1 (50.0)	0	0	1 (50.0)	0	0	0	1 (50.0)
	**Special (dry) bread (9)**	**9 (100.0)**	**0**	**1 (11.1)**	**2 (22.2)**	**0**	**0**	**0**	**0**	**0**
	Rusks (3)	3 (100.0)	0	0	1 (33.3)	0	0	0	0	0
	Breadsticks (3)	3 (100.0)	0	0	1 (33.3)	0	0	0	0	0
	Breadcrumbs (3)	3 (100.0)	0	1 (33.3)	0	0	0	0	0	0
**Total (33)**		**32 (97.0)**	**10 (30.3)**	**10 (30.3)**	**6 (18.2)**	**10 (30.3)**	**1 (3.0)**	**1 (3.0)**	**2 (6.1)**	**14 (42.4)**

Results are expressed as frequency (n: number of products including a specific ingredient) and (percentage based on the total products within the category or the subgroup)
